# Inhibition of acyl‐CoA synthetase long‐chain isozymes decreases multiple myeloma cell proliferation and causes mitochondrial dysfunction

**DOI:** 10.1002/1878-0261.13794

**Published:** 2025-01-23

**Authors:** Connor S. Murphy, Heather Fairfield, Victoria E. DeMambro, Samaa Fadel, Carlos A. Gartner, Michelle Karam, Christian Potts, Princess Rodriguez, Ya‐Wei Qiang, Habib Hamidi, Xiangnan Guan, Calvin P. H. Vary, Michaela R. Reagan

**Affiliations:** ^1^ Center for Molecular Medicine MaineHealth Institute for Research Scarborough ME USA; ^2^ University of Maine Graduate School of Biomedical Science and Engineering University of Maine Orono ME USA; ^3^ School of Medicine Tufts University Boston MA USA; ^4^ University of New England Biddeford ME USA; ^5^ Vermont Integrative Genomics Resource DNA Facility University of Vermont Burlington VT USA; ^6^ Genentech, Inc. San Francisco CA USA

**Keywords:** ACSL, cell metabolism, fatty acid, hematological malignancies, multiple myeloma, Triacsin C

## Abstract

Multiple myeloma (MM) is an incurable cancer of plasma cells with a 5‐year survival rate of 59%. Dysregulation of fatty acid (FA) metabolism is associated with MM development and progression; however, the underlying mechanisms remain unclear. Herein, we explore the roles of long‐chain fatty acid coenzyme A ligase (ACSL) family members in MM. ACSLs convert free long‐chain fatty acids into fatty acyl‐CoA esters and play key roles in catabolic and anabolic fatty acid metabolism. Analysis of the Multiple Myeloma Research Foundation (MMRF) CoMMpass^SM^ study showed that high *ACSL1* and *ACSL4* expression in myeloma cells are both associated with worse clinical outcomes for MM patients. Cancer Dependency Map (DepMap) data showed that all five ACSLs have negative Chronos scores, and *ACSL3* and *ACSL4* were among the top 25% Hallmark Fatty Acid Metabolism genes that support myeloma cell line fitness. Inhibition of ACSLs in myeloma cell lines *in vitro*, using the pharmacological inhibitor Triacsin C (TriC), increased apoptosis, decreased proliferation, and decreased cell viability, in a dose‐ and time‐dependent manner. RNA‐sequencing analysis of MM.1S cells treated with TriC showed a significant enrichment in apoptosis, ferroptosis, and endoplasmic reticulum (ER) stress, and proteomic analysis of these cells revealed enriched pathways for mitochondrial dysfunction and oxidative phosphorylation. TriC also rewired mitochondrial metabolism by decreasing mitochondrial membrane potential, increasing mitochondrial superoxide levels, decreasing mitochondrial ATP production rates, and impairing cellular respiration. Overall, our data support the hypothesis that suppression of ACSLs in myeloma cells is a novel metabolic target in MM that inhibits their viability, implicating this family as a promising therapeutic target in treating myeloma.

## Graphical abstract

Triacsin C inhibition of the acyl‐CoA synthetase long chain (ACSL) family decreases multiple myeloma cell survival, proliferation, mitochondrial respiration, and membrane potential. Made with Biorender.com.

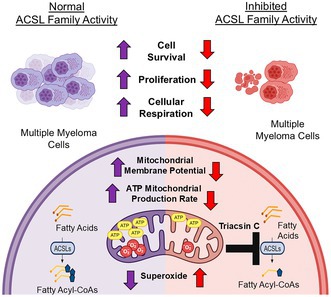

**Abbreviations**
ACSL: long‐chain fatty acid coenzyme A ligase
AML: acute myeloid leukemia
BMI: body mass index
CRC: colorectal cancer
ER: endoplasmic reticulum
FA: fatty acids
HR: hazard ratio
IMiDs: immunomodulatory drugs
ISS: International Staging System
KEGG: Kyoto Encyclopedia of Genes and Genomes
MM: multiple myeloma
MMRF: Multiple Myeloma Research Foundation
OS: overall survival
TMRE: tetramethylrhodamine ethyl ester
TriC: Triacsin C

## 1 Introduction

Multiple myeloma (MM) is an incurable cancer of plasma cells with a 59% 5‐year survival rate [1]. A hallmark of cancer cells is their ability to adapt to high energy demands through dysregulated cellular metabolism [2]. Indeed, alterations in glucose and glutamine metabolism have been shown to support myeloma cells, underscoring metabolic pathways as promising therapeutic targets [3]. However, pursuing fatty acid (FA) metabolism as a novel anti‐myeloma avenue has only been preliminarily explored [4, 5, 6, 7, 8].

Recent work on other cancers shows promise for targeting members of the acyl‐coenzyme A long‐chain synthetase (ACSL) family of proteins, which activates long‐chain fatty acids (saturated and unsaturated FAs with chain lengths of 8–22 carbons) into fatty acyl‐CoA esters, which can then be used for catabolic or anabolic metabolism (reviewed in [9]). In the catabolic pathway, fatty acid oxidation (FAO) in the mitochondria generates ATP, whereas anabolically, FAs comprise the substrates needed to synthesize triacylglycerols (TAGs, used to store energy), phospholipids, and other cell and organelle membrane lipids. System‐level analysis of ACSLs across cancer types has revealed that ACSL expression levels and their roles as oncogenes or tumor suppressors are heterogeneous and cancer type dependent [10]. While ACSL1 has been shown to support the proliferation of both colorectal cancer (CRC) and breast cancer (BC) cell lines, other evidence implicates ACSL1 as a tumor suppressor in non‐squamous cell lung carcinoma (NSCLC) cells [10, 11]. Additionally, ACSL1 and ACSL4 support invasion of CRC, prostate cancer, and quadruple‐negative BC cells, and enhance glycolysis [11, 12, 13, 14, 15]. In estrogen receptor‐positive BC, ACSL4 modulates drug efflux pumps to support chemotherapy resistance [13]. ACSLs also regulate metabolism in a cancer type‐specific manner: ACSL3 drives FAO in opposing directions in BC and NSCLC, while supporting proliferation in both cancer types [16, 17]. Therefore, it is critical to understand the roles of ACSLs in specific cancer contexts.

ACSLs play important roles in the development of lymphoid hematological malignancies. In a retrospective study of leukemia patients, *ACSL6* expression was positively correlated with overall survival (OS), suggesting that ACSL6 may be a tumor suppressor in leukemia [10]. In that analysis, of all ACSLs, only *ACSL4* was shown to be overexpressed in myeloma cells relative to control tissues [10]. Zhang *et al*. [8] also reported that *ACSL4* was overexpressed in primary MM cells and supported MM cell proliferation, possibly through the c‐Myc/sterol regulatory element binding protein (SREBP) axis. These investigators also showed that *ACSL4* expression in MM cells was positively correlated with sensitivity to ferroptosis, an iron‐dependent form of programmed cell death [8]. Despite these studies, the contribution of the ACSL family to myeloma cell fitness remains largely unaddressed.

Triacsin C (TriC) is a bacterial alkenyl‐N‐hydroxytriazene metabolite and a competitive inhibitor of ACSL activity that targets their FA‐binding domain [18]. TriC has been shown to inhibit the growth of human BC cells [19, 20], CRC cells [14], and acute myeloid leukemia (AML) cells, and can synergize with other anti‐cancer therapies [21]. TriC also induces apoptosis in glioma cells and synergizes with the apoptosis inducer etoposide, causing substantial cytotoxicity in glioma cells both *in vitro* and *in vivo* [22]. However, endometrial cancer cells exhibited elevated resistance to TriC [23]. Overall, these data suggest that TriC is a safe, novel anti‐cancer agent that could be beneficial in multiple myeloma preclinically, and could serve as the basis for a new clinical therapeutic direction.

## 2 Materials and methods

### 2.1 MM patient survival analysis: clinical single cell and bulk RNA‐sequencing data

Bone marrow biopsies from newly diagnosed MM patients (*N* = 754) in the MMRF CoMMpass^SM^ study (NCT01454297), version IA23, were collected, enriched by CD138 positive bead selection and subjected to bulk RNA‐seq analysis; this work, the consent process, and the oversight committees have been previously described [24]. The study methodologies conformed to the standards set by the Declaration of Helsinki; experiments were undertaken with the understanding and written consent of each subject; the study methodologies were approved by the local ethics committee. These data were analyzed as follows: patients were binarized into high or low groups based on the median cut‐off for each *ACSL* family member. Cox regression examined associations between each gene and time to event [overall survival (OS)] and time to second‐line treatments (progression). Each Hazard Ratio (HR) was calculated comparing patients above to below median for each gene. Significant associations were visualized by a Kaplan–Meier curve analysis.

To profile the tumor microenvironment, CD138 negative samples were subjected to scRNA‐seq by the MMRF Immune Profiling Consortium; details about sample inclusion, library construction and preprocessing were described previously [25, 26]. Briefly, the scRNA‐seq fastq reads were aligned to the human transcriptome (GRCh38) to obtain the gene‐barcode count matrix using the Cell Ranger pipeline with no intron inclusion (10x genomics, v7.0.1, 10X Genomics Inc, Pleasanton, CA). For a subset of samples with mouse 3T3 cells spike‐in, the reads were aligned to the human plus mouse transcriptomes (mm10). Initial cell quality control included the following filtering: human gene counts percentage > 90% to remove mouse spike‐in cells, number of UMIs (lower: 800; upper: 50 000), number of genes (lower: 300; upper 7500), and mitochondrial contents (< 20%). The preprocessed count matrices were aggregated (1 099 695 cells) and subject to the standard Seurat analysis pipeline (v3.2.2) [27]. After clustering, cells with high hemoglobin gene contents (> 1%) and clusters co‐expressing multiple major cell lineage markers (T/NK cell, B and plasma cell, myeloid cell, and red blood cell) were further removed, yielding an object of 922 740 cells across 321 samples where 233 samples were collected from the baseline. Finer cell type annotation was performed using azimuth (v0.4.5) [28] using the built‐in human bone marrow reference [29, 30, 31]. Next, a pseudo‐bulk expression matrix was generated by averaging the normalized gene expression across cell types and samples, and only the 233 baseline samples were used for subsequent analysis. Cell type‐specific expression was determined for each ACSL family member. As before, the patients were binarized into high or low groups based on the median cut‐off. Cox regression examined associations between cell type‐specific gene expression and time to event. Hazard Ratios were calculated comparing patients above to below median for each cell type. Significant associations were visualized by a Kaplan–Meier curve.

### 2.2 Cancer dependency map analysis

The gene fitness scores (Chronos scores) [32] for a modified list of the Hallmark Fatty Acid Metabolism genes (GSEA M5935 https://www.gsea-msigdb.org/gsea/msigdb/cards/HALLMARK_FATTY_ACID_METABOLISM, Table S1) in 21 human MM cell lines from the Cancer Dependency Map (DepMap) dataset (https://depmap.org/portal/download/) were reported. Human MM cell line gene and protein expression data were downloaded from the Cancer Dependency Map/Cancer Cell Line Encyclopedia (Expression 22Q2_Public) [33].

### 2.3 Cell lines and culture conditions

MM.1S (ATCC; RRID:CVCL_8792), RPMI‐8226 (ATCC; RRID:CVCL_0014), and OPM‐2 (DSMZ; RRID:CVCL_1625), U266B (ATCC; RRID:CVCL_0566), and MM.1R (MM.1R; CVCL_8794) cells, as well as luciferase‐ and GFP‐expressing MM.1S (CVCL_8792), MM.1S^gfp+/luc+^, generously provided by Dr. Irene Ghobrial, were cultured as previously described [4]. Briefly, cells were cultured in RPMI‐1640 basal media supplemented with 10% (15% for U266B1 cells) fetal bovine serum (FBS, VWR) with 1% antibiotic‐antimycotic (ThermoFisher Scientific, Cat # 15240112) at a cell density of 4 × 10^5^ cells·mL^−1^ in tissue culture‐treated T‐75 flasks (VWR) [4]. MM.1S^gfp+/luc+^ cells were used for experiments involving MM.1S cells unless otherwise stated. Human myeloma cell lines were authenticated with genomic DNA isolated with the QIAamp DNA Mini Kit (Qiagen, Hilden, Germany), and short tandem repeat (STR) analysis was performed with the CLA IdentiFiler™ Plus PCR Amplification Kit (ThermoFisher Scientific) and ABI SeqStudio Genetic Analyzer (ThermoFisher Scientific) according to the manufacturer's protocol through the Vermont Integrative Genomics Resource at the University of Vermont. STR profiles were compared between the experimental results and the reference using the Cellosaurus STR Similarity Search tool with the Tanabe algorithm, scoring only non‐empty markers and excluding amelogenin (clastr v1.4.4, Swiss Institute of Bioinformatics, Geneva, Switzerland). Experiments were performed with mycoplasma‐free cells and authenticated or purchased new at least every 3 years.

### 2.4 Triacsin C treatment

Triacsin C (TriC) was purchased from Cayman Chemical (Ann Arbor, MI, USA). MM.1S^gfp+/luc+^, MM.1R, OPM‐2, RPMI‐8226, and U266B1 cells were seeded into either tissue culture‐treated white bottom 96 well plate (4.33 × 10^4^ cells per well), tissue culture‐treated 24‐well plates (1 × 10^5^ cells per well), tissue culture‐treated 6‐well dishes (4.81 × 10^5^ cells per well), or tissue culture‐treated T‐25 flasks (2 × 10^6^ cells per flask; Avantor/VWR, Cat. No. 10861‐568) under the growth conditions described above. MM cells were treated with TriC or dimethyl sulfoxide (DMSO, vehicle). Samples were collected at 24‐h intervals and subjected to functional analyses below.

### 2.5 Myeloma cell quantification, viability, and apoptosis

For quantification and viability testing, MM cells were collected and resuspended in RPMI‐1640 + 10% FBS + 1% antibiotic‐antimycotic, and diluted 1 : 1 in 0.4% Trypan Blue. Viable and non‐viable cells were counted using a hemocytometer. To characterize apoptosis, MM cells were collected, washed 3 times with Cell Staining Buffer (BioLegend, Cat. No. 420201, San Diego, CA, USA) and stained with APC‐Annexin V (1 : 20, BioLegend Cat. no. 640920), DAPI (0.004 μg·mL^−1^, ThermoFisher, Cat. No. D1306) in Annexin V Binding Buffer (BioLegend, Cat. no. 422201) for 15 min at room temperature. An initial gate was made in the FSC‐A vs. SSC‐A and doublets were excluded comparing FSC‐A vs. FSC‐H. Within the single cell gate, positive populations were identified by comparing stained and unstained samples. For all flow cytometric analyses, a minimum of 1 × 10^4^ events were collected per sample on a MACSQuant Analyzer (Miltenyi Biotec, Bergisch Gladbach, Germany) and analyzed using flowjo v.10 (Becton, Dickinson & Company, Ashland, OR, USA).

### 2.6 Human peripheral blood mononuclear cells (PBMC) isolation and determination of viability after Triacsin C treatment

Human PBMCs from deidentified healthy donors provided by Drs. Sergey Ryzhov and Douglas Sawyer were collected from April 2013 to April 2014 as part of a previous study approved by the Institutional Review Board at Vanderbilt University with all donors giving written informed consent [34]. Study methodologies conformed to the standards set by the Declaration of Helsinki. PBMCs were isolated from venous blood of healthy donors using BD Vacutainer EDTA tubes (Becton Dickinson) and isolated on a Ficoll‐Paque Premium gradient (GE Healthcare Life Sciences/Cytivia, Cat. No. 17544202, Marlborough, MA, USA) within 4–6 h of drawing. Cells were frozen at −80 °C in Fetal Calf Serum : DMSO, 9 : 1 and subsequently stored in liquid nitrogen. Upon thawing, cell viability was determined by mixing the cell suspension and Cellometer ViaStain™ Acridine Orange/Propidium Iodide Viability Stain (Nexcelom Bioscience, CS2‐0106‐5ML, Lawrence, MA, USA) 1 : 1 and reading viability on the Cellometer K2 (Revvity, CMT‐K2‐MX‐150, matrix software v2.1.7.6, Waltham, MA, USA) or trypan blue staining as described above. PBMCs from three healthy donors were plated in tissue culture‐treated 24‐well plates (1 × 10^5^ cells per well) in RPMI‐1640 + 10% FBS + 1% antibiotic‐antimycotic +20 ng·mL^−1^ recombinant human colony stimulating factor 2 (CSF2, carrier‐free, Biolegend, Cat. No. 572902) and treated with Triacsin C or vehicle. After 48 h of incubation, cells were washed 3 times with PBS (VWR, Cat. No. 02‐0119‐1000) and a fifth of the total cell volume was loaded into non‐tissue culture‐treated 96‐well round‐bottom plates (Thermo‐Fisher Scientific, Cat. No. 268152). Propidium Iodide (Miltenyi Biotec, Cat. No. 130‐093‐233) was added to each sample 1 : 100 and samples were run on a MACSQuant® VYB (Miltenyi Biotec). Cell count and viability was determined using MACSQuant® Analyzer software (v2.11.1907.19925).

### 2.7 Assessment of changes in redox potential in PBMCs

1.6 × 10^3^ PBMCs from three healthy human donors isolated from the same study as above were plated in tissue culture‐treated white flat‐bottom 96 well plates (ThermoFisher Scientific, 136102) in RPMI‐1640 + 10% FBS + 1% antibiotic‐antimycotic +20 ng·mL^−1^ recombinant human Colony stimulating factor 2 (CSF2, carrier‐free, Biolegend, Cat. No. 572902) and treated with various concentrations Triacsin C or vehicle (DMSO) as above but with a 1× final concentration of the RealTime Glo™ MT Cell Viability Assay (Promega, Cat. No. G9711, Madison, WI, USA) reagents: MT Cell Viability substrate and NanoLuc® luciferase. Before the initial reading at time zero, samples were incubated at room temperature under light‐protected conditions for 10 min then read on a Promega Glomax Explorer (Promega, GM3500). Plates were incubated at 37 °C + 5% CO_2_ and luminescence was read at 24‐h intervals for 72 h.

### 2.8 Intracellular characterization of BAX protein

MM.1S cells were washed three times with Cell Staining Buffer (BioLegend) and then fixed in 1× Fixation Buffer (4% paraformaldehyde, BioLegend). Cells were washed 3 times with Cell Staining Buffer and stained with either Alexa Fluor (AF) 488 mouse anti‐human BAX antibody (0.5 μg·mL^−1^, Biolegend, Cat. No. 633603) or AF488 Mouse IgG1 κ isotype control (5 μg·mL^−1^, Biolegend, Cat. No. 400129) in 1× Intracellular Staining Permeabilization Wash Buffer (Perm/Wash, BioLegend, Cat. No. 421002) for 15 min at room temperature. Cells were washed 2 times in 1× Perm/Wash buffer and resuspended in Cell Staining Buffer (Biolegend) prior to flow cytometry analysis. A total of 2 × 10^4^ events were collected using a MACSQuant (Miltenyi Biotec) and analyzed using flowjo v10.6.1. Data are presented as the mean fluorescence intensity (MFI) of the FITC‐H channel within the FSC‐A and SSC‐A gates.

### 2.9 Myeloma cell cycle and Ki‐67 staining

MM cells were washed three times with Cell Staining Buffer (BioLegend) and fixed in 1× Fixation Buffer (4% paraformaldehyde, BioLegend). Cells were washed three times with Cell Staining Buffer and stained with Alexa Fluor 647 anti‐human Ki‐67 antibody (1 : 100) and DAPI (0.5 μg·mL^−1^) respectively in 1× Intracellular Staining Permeabilization Wash Buffer (BioLegend). The cells were resuspended in cell staining buffer (BioLegend, Cat. No. 420201) prior to flow cytometry using a MACSQuant Analyzer (Miltenyi Biotec). Initial gates were made in the FSC‐A vs. SSC‐A and doublets were excluded using FSC‐A vs. FSC‐H. In the same sample, both DAPI and Ki67‐AF647 was analyzed, positive populations were identified by comparing stained and unstained samples. A minimum of 10 000 events were collected per sample.

### 2.10 Flow cytometric characterization of mitochondrial number/mass, mitochondrial membrane potential, and mitochondrial superoxide levels

MM cells were washed three times with Cell Staining Buffer (BioLegend) and resuspended in their respective cell culture media with 100 nm MitoTracker Green (Invitrogen, Cat. No. M7514), and incubated for 30 min at 37 °C. Cells were washed three times with cell staining buffer and resuspended in Cell Staining Buffer (BioLegend) prior to flow cytometry using a MACSQuant Analyzer (Miltenyi Biotec). To characterize mitochondrial membrane potential, MM cells were washed three times with Cell Staining Buffer (BioLegend) and resuspended in tetramethylrhodamine ethyl ester (TMRE) buffer (Cayman Chemical, Ann Arbor, MI, USA) containing 100 nm TMRE (Cayman Chemicals, Cat. no. 701310) and incubated for 30 min at 37 °C. Cells were pelleted and resuspended in TMRE buffer and subjected to flow cytometry on a MACSQuant Analyzer (Miltenyi Biotec).

### 2.11 Assessment of lipid peroxidation and mitochondrial reactive oxygen species (ROS)

To measure lipid peroxidation, MM.1S and OPM‐2 cells were treated with Triacsin C (2 μm; Sigma, Cat. No. T4540‐1MG) for 24–72 h, stained in their culture media with BODIPY 581/591 (10 μm; ThermoFisher, Cat. No. D3861) for 30 min at 37 °C, and washed with Cell Staining Buffer (BioLegend, Cat. No. 420201) to remove excess label, and analyzed for their red and green signal on a MACSQuant Analyzer (Miltenyi Biotec) according to the manufacturer's protocol. The same cells were also assessed for viability with RealTime‐Glo MT (Promega, Cat. No. G9713) per the manufacturer's instructions.

To measure mitochondrial superoxides, MM.1S cells were treated with TriC (Sigma‐Aldrich, Cat. No. T4540‐1MG, Burlington, MA, USA) at 0–3 μm for up to 72 h. At 24, 48, and 72 h, cells were stained in their culture media with MitoSox Green (1 μm; ThermoFisher, M36006) for 30 min at 37 °C and washed with Cell Staining Buffer (Biolegend) to remove excess label. Cells were resuspended in Cell Staining Buffer (Biolegend) prior to flow cytometry via MACSQuant Analyzer (Miltenyi) using manufacturer recommended settings.

### 2.12 Cellular metabolic analysis

5 × 10^6^ MM.1S^gfp+/luc+^ cells were treated with DMSO or 1 μm TriC for 24 h in T‐25 flasks (Avantor/VWR; Cat. no. 10861‐568). Cells were then harvested, centrifuged, and resuspended in XF‐DMEM media (pH 7.4; Agilent Technologies, Cat # 103575‐100, Santa Clara, CA, USA) containing 1 mm sodium pyruvate, 10 mm glucose and 2 mm glutamine prior to plating on Seahorse XF 96 PDL‐coated plates (Agilent, Cat # 103730‐100) at a density of 75 000 cells per well per the manufacturer's instructions. Mitochondrial function was determined using a Mitochondrial Stress Test on the Seahorse XFe96 analyzer (Agilent Technologies), as previously described [4]. Cells were also analyzed for total, mitochondrial, and glycolytic ATP production rates using a Seahorse XF ATP Production Rate Assay according to the manufacturer's instructions. The data presented here are representative of at least three independent experiments with ≥ 24 wells per treatment.

### 2.13 Acyl‐CoA synthetase long‐chain activity assay

This protocol was adapted from Nchoutmboube *et al*. [35]. MM.1S cells were plated in 6 well dishes (4.81 × 10^5^ cells per well) in RPMI + 0.5% Fatty Acid (FA) Free BSA + 1% antibiotic‐antimycotic and incubated with 0.5 μm fluorescent fatty acid substrate BODIPY FL C_16_ at 37 °C for 2 h. The cells were incubated with TriC or DMSO for 2 h, collected, washed 3 times with 0.2% FA‐free BSA/PBS (ThermoFischer, Cat. No. AAJ6494422) to remove excess label, and subjected to n‐heptane extraction to separate the product (BODIPY FL C_16_) from the substrate (BODIPY FL C_16_‐CoA) as follows. Cells were resuspended in 8.5% sucrose +0.5 μm EDTA +10 mm Tris Buffer (pH 8.0) + 0.1% Triton X‐100 and incubated at room temperature for 25 min. Lysates were centrifuged for 10 min at 14 000 ***g***, and the supernatant was transferred to fresh tubes. Heptane (Cat. no. 34873, Millipore Sigma, Burlington, MA, USA) was added to the supernatant (1 volume supernatant: 6 volumes of n‐heptane), shaken at 1300 rpm for 10 min, and centrifuged for 5 min at 12 000 ***g***. n‐Heptane was removed using a pipette, and the aqueous layer was subsequently extracted with n‐heptane 3 more times. The aqueous layer was read on a black 96‐well plate (Corning Cat No. 3603, 475 nm/500‐525 nm ex/em, Corning, NY, USA).

### 2.14 Total mRNA extraction and quantitative real‐time polymerase chain reaction (qRT‐PCR)

Total RNA was harvested in QIAZOL and prepared using the Qiagen miRNEASY Kit with DNase On‐column digestion (Qiagen), according to the manufacturer's protocol. mRNA was quantified and tested for quality and contamination using a Nanodrop 2000 (Thermo Fisher Scientific) and subjected to quality control minimum standards of 260/230 > 2.0 and 260/280 > 1.8 before downstream applications. For qPCR, cDNA was synthesized using MultiScribe reverse transcriptase (High‐Capacity cDNA, Applied Biosciences, ThermoFisher Scientific) according to the manufacturer's instructions using 500 ng of total RNA. Relative transcript expression was determined using SYBR Master Mix (Bio‐Rad, Hercules, CA, USA) and thermocycling reactions on a CFX‐96 or Opus system (Bio‐Rad) using 500 ng of cDNA. Target transcripts (Tables S2 and S3) were normalized to *TATA‐box binding protein* (*TBP*) using the $2^{-\Delta \Delta C_{t}}$ method. Data were analyzed using Bio‐Rad CFX Manager 3.1.

### 2.15 RNA sequencing sample preparation and analysis

A total of 5 × 10^6^ MM.1S^gfp+/luc+^ cells were seeded in T‐25 flasks (Avantor/VWR, Cat. No. 10861‐568) and treated with the vehicle (DMSO) or 1 μm TriC for 24 h. Replicates were defined as MM.1S^gfp/luc+^ cells of the same passage grown in parallel. After 24 h, RNA was isolated using a Qiagen RNeasy Plus Mini Kit (Qiagen) Cat. no. 74136, according to the manufacturer's protocol. Samples were evaluated on Bioanalyzer BA210O RNA pico chips and quantified using the Qubit HS DNA reagent. Sequence libraries were prepared with the Takara Pico V2 library prep using 6 ng of total RNA and sequenced with an Illumina HiSeq 1500/2500. RNA‐sequence data were analyzed using the nf‐core/rnaseq pipeline v3.9 [36, 37] using the nextflow workflow manager v22.10.2 [38]. Raw reads were subjected to quality checking and reporting (fastqc v0.11.9/multiqc v1.13) [39], and low‐quality sequence data (Phred score < 20) were removed using trim galore v 0.6.7 [40]. The reads were aligned to the *Homo sapiens* hg38 reference genome using star v2.7.10a [41] and samtools v1.15.1 [42]. Read counts were quantified using salmon v1.5.2 [43], and deseq2 v1.28.0 [44] was used to identify differentially expressed genes (DEGs) using a cut‐off value of (log_2_(FC) > |1|, q‐value < 0.05) using the Wald test and adjusted for multiple testing using the Benjamini and Hochberg method. Gene ontology enrichment analysis was performed using the Enrichr package and STRINGv11, with a high confidence score cut‐off of 0.70. Enrichr was used to identify significantly enriched Reactome [45] and KEGG [46] pathways within these DEGs.

### 2.16 Sample preparation for mass spectrometry proteomics of TriC‐treated MM.1S Cells

MM.1S^gfp+/luc+^ cells were treated with vehicle (DMSO) or 1 or 2 μm TriC for 48 h, collected, washed 3× with cold PBS, and flash‐frozen. Cells were then solubilized in ice‐cold RIPA buffer, and DNA was sheared using a probe‐tip sonicator (Branson Ultrasonifier 250, Branson Ultrasonic Corporation, Danbury, CT, USA, 3 × 10 s). Each sample was then centrifuged (14 000 ***g***) at 4 °C and the supernatant was collected. Protein preparations for liquid chromatography and mass spectrometry were essentially as reported [47]. One hundred micrograms of protein was taken from each sample and reduced using 5 mm TCEP (tris(2‐carboxyethyl)phosphine hydrochloride; Strem Chemicals, Newburyport, MA, USA). The reaction was allowed to proceed for 20 min at 56 °C and alkylated for 30 min in the dark with 10 mm iodoacetamide at room temperature (G‐Biosciences, St. Louis, MO, USA). Proteins were precipitated at −20 °C with ethanol, and pellets were washed twice with ice‐cold ethanol followed by overnight incubation at 37 °C in 100 mm ABC containing 1 mm CaCl_2_ and trypsin (Sequencing grade, modified, Promega Co, Madison, WI, USA). Digested proteins were evaporated and each sample was freed from salts and buffers by solid‐phase extraction on C18 resin using cartridges prepared in‐house. Briefly, for each sample, a C18 StageTip was prepared according to the previously reported procedure [48]. Octadecyl‐derivatized silica (4 mg; SiliaSphere PC, C18 monomeric, 25 μm particles, 90 Å pore size, SiliCycle Inc., Québec City, QC, Canada) suspended in LC–MS‐grade isopropanol (Honeywell, Morris Plains, NJ, USA) was added to each tip. Each cartridge was then equilibrated, and the samples were purified according to the StageTip protocol referenced above. Purified peptides were eluted directly into autosampler vials for use in LC–MS instrumentation using 100 μL elution buffer, and the solvent was removed by vacuum centrifugation. Each sample was then resuspended in a volume of sample LC/MS loading solvent [5% formic acid (Optima grade, Thermofisher Scientific) and 4% acetonitrile (both water and acetonitrile were LC–MS‐grade, Honeywell)] to yield an approximate concentration of 1 μg·μL^−1^ peptides.

### 2.17 Mass spectrometry proteomics of TriC‐treated MM.1S Cells

Chromatography, mass spectrometry, and data analysis were performed as previously described [49]. Key differences between the protocols are highlighted here. Sample separation was performed on an Eksigent NanoLC 425 nano‐UPLC System (Sciex, Framingham, MA, USA) in direct‐injection mode with a 5 μL sample loop made in‐house. The analysis was performed in positive ion mode on a TripleTOF 5600 quadrupole time‐of‐flight mass spectrometer (Sciex).

### 2.18 Statistical analysis

All graphs were created using graphpad prism, boston, ma, usa (v9 or above); statistical significance was determined using one‐way or two‐way ANOVA with Tukey's, Šídák's, or Dunnett's multiple comparisons tests, Student's *t*‐test, or Welch's test unless otherwise stated. Data represent the mean ± standard deviation, unless otherwise noted. Significance is indicated as: *P < 0.05; **P < 0.01; ***P < 0.001; ****P < 0.0001.

## 3 Results

### 3.1 Targeting the long‐chain acyl‐CoA Synthetases (ACSL) family is clinically relevant in multiple myeloma

**Fig. 1:**
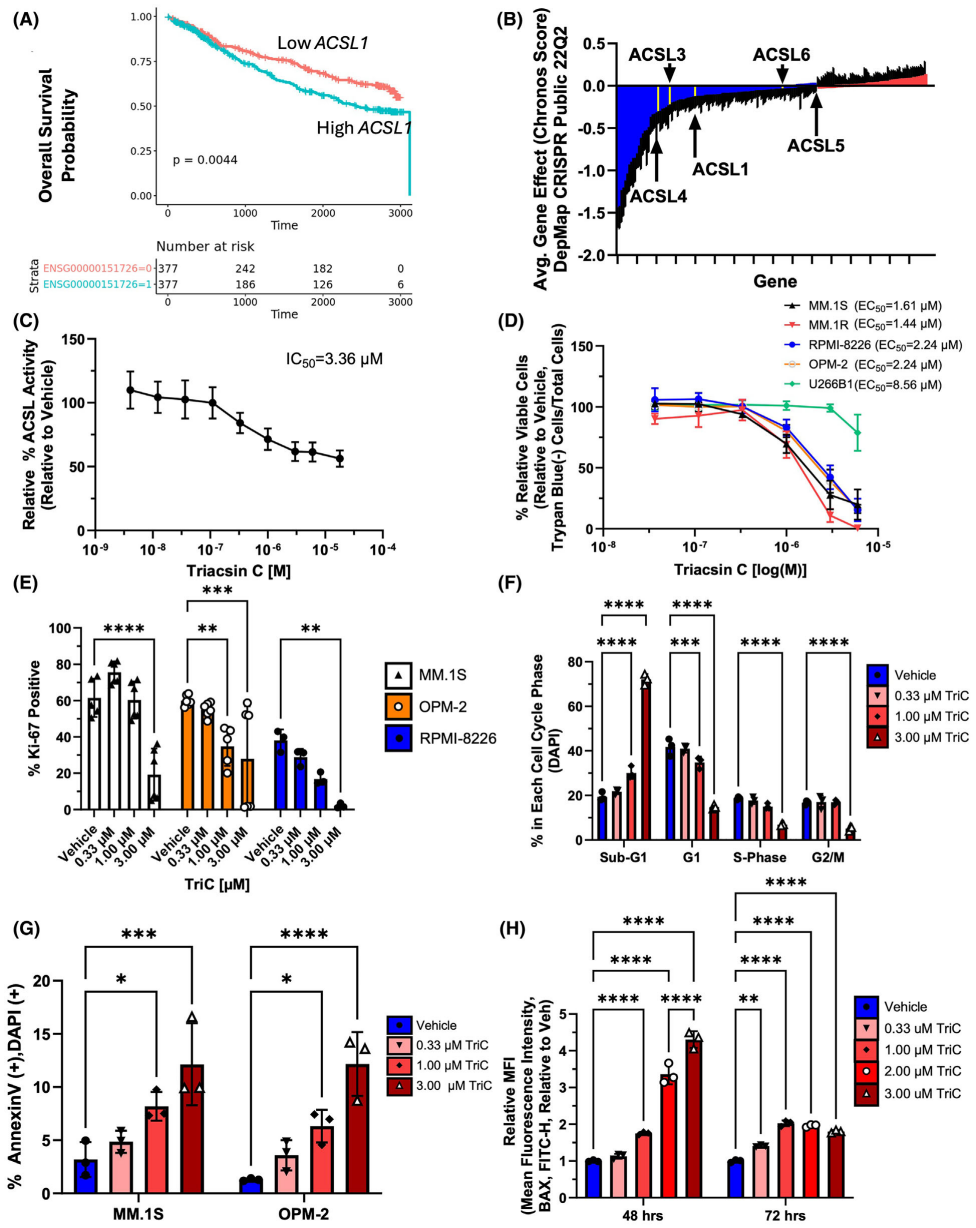
Targeting the ACSLs is a Novel Target to Inhibit Myeloma Cell Proliferation and Survival. (A) Overall survival of patients from CoMMPass trial with high or low *ACSL1* expression (relative to median, ENSG00000151726) for all patients (*n* = 754). *P*‐values are calculated from log‐rank Kaplan–Meier test. (B) Hallmark Fatty Acid Metabolism genes from Gene Set Enrichment Analysis (M5935) displayed as the average gene fitness (Chronos Score) data from the Cancer Dependency Map of 21 human myeloma cell lines. Blue bars represent targets of interest with average Chronos scores < 0 (fitness defect upon CRISPR knockout), while red bars are Chronos scores > 0. ACSL family members are highlighted in yellow. (C) ACSL activity in MM.1S cells after treatment with a range of Triacsin C (TriC) doses; IC_50_ values were calculated using non‐linear regression (four parameter, variable slope) based on the relative ACSL Activity to the vehicle; *n* = 3. (D) MM.1S, MM.1R, RPMI‐8226, OPM2 and U266B1 cells were incubated with various doses of TriC for 48 h and stained with Trypan Blue to quantify viable cells·mL^−1^. EC_50_ values were calculated using non‐linear regression (four parameter, variable slope) in graphpad prism v9.4.1. *n* = 3. (E) Proliferation of human myeloma cell lines MM.1S, OPM‐2 and RPMI‐8226 treated with various doses of TriC for 48 h and stained with AF647 anti‐human Ki67 (% positive); MM.1S (*n* = 5), OPM‐2 (*n* = 6), RPMI‐8226 (*n* = 3). (F) Cell cycle distribution of MM.1S cells treated with various doses of TriC for 48 h and stained with DAPI; *n* = 3. (G) Apoptosis assay using Annexin V/DAPI staining of MM.1S and OPM2 cells treated with various doses of TriC for 48 h; *n* = 3. (H) Intracellular BAX (AF488 mouse anti‐human BAX antibody) levels in MM.1S cells treated with various doses of TriC for 48h or 72h; data displayed as FITC MFI; *n* = 3. Statistics: Two‐way ANOVA with Tukey's multiple comparison test (E–G) or Šídák's multiple comparisons test (H). All data are mean ± SD, *P < 0.05, **P < 0.01, ***P < 0.001 ****P < 0.0001.

To determine the clinical significance of targeting ACSLs in myeloma, we first explored the MMRF's CoMMpass^SM^ bulk RNA‐seq data of CD138^+^ cells from 754 MM patients at baseline. *ACSL3*, *ACSL4*, and *ACSL5* were highly expressed at roughly the same level and with the same distribution across patients; *ACSL1* was moderately expressed with a wide expression distribution; and *ACSL6* was essentially not expressed (median TPM was < 0.5) (Fig. S1). For all expressed ACSLs, we then examined how their levels associate with the following clinical outcomes: overall survival (OS), time to second line of treatment, and time to second‐line treatment only (progression) in patients whose first line of therapy was combined bortezomib/IMIDs‐based (to remove confounding factors affected by different first‐line treatment modalities). Neither tumor *ACSL3* nor *ACSL5* expression were associated with clinical outcome (data not shown). However, low expression of *ACSL1* did correlate with improved OS (Fig. 1A), and this held true even when performing a multivariate cox regression analysis accounting for age, BMI, International Staging System (ISS) value, and gender (Fig. S2). Interestingly, low *ACSL4* expression also correlated with better OS, and with longer time to second‐line therapy, and longer time to second‐line therapy for those patients combined bortezomib/IMIDs‐based first‐line therapy (Fig. S3). After multivariate analysis, this OS benefit remained when accounting for BMI or age alone, but when accounting for all standard variables (age, BMI, ISS value, and gender), *ACSL4* expression no longer significantly associated with survival, demonstrating that its association may be confounded by other risk and gender factors. Overall, these data further support the idea that the ACSLs, especially ACSL4 and ACSL1, have associations with clinical outcome in newly‐diagnosed MM patients treated with proteasome inhibitor and/or IMiDs, and may be tested as novel targets. Finally, to determine potential off‐target effects of *ACSL* inhibition in bone marrow (BM) microenvironment cells, we analyzed myeloma patient survival in relation to expression of *ACSL* expression in the CD138^‐^ BM cells from the CoMMpass dataset using single cell RNA‐sequencing data. We found only 4 significant results in this analysis: First, low *ACSL3* expression in Naïve B cells, and low *ACSL5* and *ACSL6* in Naïve CD8 cells, correlated with worse patient survival outcomes, demonstrating a potential reliance on these for an effective anti‐cancer immune response (Fig. S4). Secondly, high *ACSL5* expression in promegakaryocytes correlated to worse outcomes. These data suggest that specific targeting of ACSL4 in the tumor environment may enable more specific effects on myeloma cells with a sparing of immune side effects, and demonstrate the complex nature of lipid metabolism influences on immune cell function in cancer.

### 3.2 Pharmacological inhibition of the ACSL family decreases multiple myeloma cell proliferation and survival

To test the hypothesis that fatty acid metabolism supports myeloma cell proliferation and survival, we investigated the Cancer Dependency Map (DepMap) [32], a collection of fitness scores of a set of human myeloma cell lines containing CRISPR/Cas9 knockouts from the Hallmark Fatty Acid Metabolism gene set (Molecular Signature Database: M5935) [50]. All of the five human ACSL family members had negative Chronos scores (suggesting they support myeloma cell line growth and survival), and *ACSL3* and *ACSL4* were in the top 25% most essential fatty acid metabolism‐related genes (Fig. 1B, Table S1). The average gene expression of *ACSL1*, *3*, *4* and *5* ranged from 3.78–5.19 TPM + 1 in myeloma cell lines but *ACSL6* was essentially not expressed (Fig. S5A). However, analysis of the DepMap protein database showed expression of all ACSL family members in six human myeloma cell lines (Fig. S5B), suggesting that inclusively targeting the ACSL family may be more advantageous than targeting ACSL isoforms independently, although the decreased specificity of this approach could lead to increased toxicity issues.

We next used Triacsin C (TriC), a small molecule inhibitor of ACSL 1, 3, 4, and 5 [51] to test the hypothesis that the ACSL family supports myeloma cells *in vitro*. We performed an enzyme activity assay [35] to determine the total cellular ACSL of TriC on MM.1S cells and found the IC_50_ to be ~ 3.36 μm (Fig. 1C). To test the hypothesis that TriC would decrease myeloma cell proliferation and survival, human MM cell lines (MM.1S, MM.1R, RPMI‐8226, OPM‐2, and U266B1) were treated with TriC (0.0366–6.00 μm) at 24‐h intervals for up to 72 h and subjected to trypan blue staining to assess cell viability. Significant differences in MM cell viability were first observed after 48 h of TriC with most human MM cells responding with a dose‐dependent decrease in viability (average EC_50_ : 1.88 μm), except for U266B1 cells (EC_50_ : 8.56 μm) (Fig. 1D). Time‐dependent and dose‐dependent decreases in viability were also observed in MM.1S cells (Fig. S5C). No effects of TriC on *ACSL* gene expression were observed (Fig. S5D).

To test for cytotoxic and cytostatic effects of TriC, we measured myeloma cell proliferation (Ki‐67 staining), cell cycle (DAPI staining), and apoptosis (Annexin V/DAPI staining), and expression of the pro‐apoptotic protein, BAX protein. At 48 h, MM.1S, OPM‐2 and RPMI‐8226 cells showed dose‐dependent reductions in Ki‐67^+^ cells in response to TriC (Fig. 1E, Fig. S6). Treatment with 1 and 3 μm TriC also significantly increased the sub‐G_1_ population and decreased G_1_ and G_2_/M populations in MM.1S, OPM‐2, and RPMI‐8226 cells (Fig. 1F; Figs S5E,F and S6). There was also a dose‐dependent increase in “late apoptotic” [Annexin V^+^/DAPI^+^] cell populations at 48 and 72 h post‐TriC in MM.1S and OPM‐2 cells (Fig. 1G), and an increase in early‐apoptotic [Annexin V^+^/DAPI^−^] cells in MM.1S, with no differences in dead [Annexin V^−^/DAPI^+^] cells, likely due to their degradation (Figs S5G,H and S7A). Lastly, we observed dose‐dependent increases in the pro‐apoptotic protein BAX in MM.1S cells treated with TriC at 48 and 72 h (Fig. 1H; Fig. S7B). Taken together, these data demonstrate that TriC decreases viability and proliferation, alters cell cycle progression, and induces apoptosis in myeloma cell lines.

To test TriC's effects on healthy cells, human peripheral blood mononuclear cells (PBMCs) from three healthy donors were treated with TriC or vehicle for 48 h and counted to measure viability, or assessed with RealTime‐Glo™ MT. By cell counting, we observed a slightly greater viability of PBMCs, compared to myeloma cells, in response to TriC, with an EC_50_ of ~ 6 μm, compared to 1.44–2.24 μm for most myeloma lines tested. Even with 3 μm TriC, there was 69% PBMC viability at 48 h (Fig. S8A). However, by RealTime‐Glo™ MT, which assesses both viability and metabolic activity, TriC‐induced dose‐ and time‐dependent decreases similarly in PBMCs, MM.1S cells, and OPM‐2 cells (Fig. S8B–D). Taken together, although further refinement and pharmaceutical development will be needed, these data suggest the ACSL family may still be a feasible and not prohibitively toxic target for MM.

### 3.3 Triacsin C induces transcriptional changes associated with cell death and the integrated stress response in myeloma cells

**Fig. 2:**
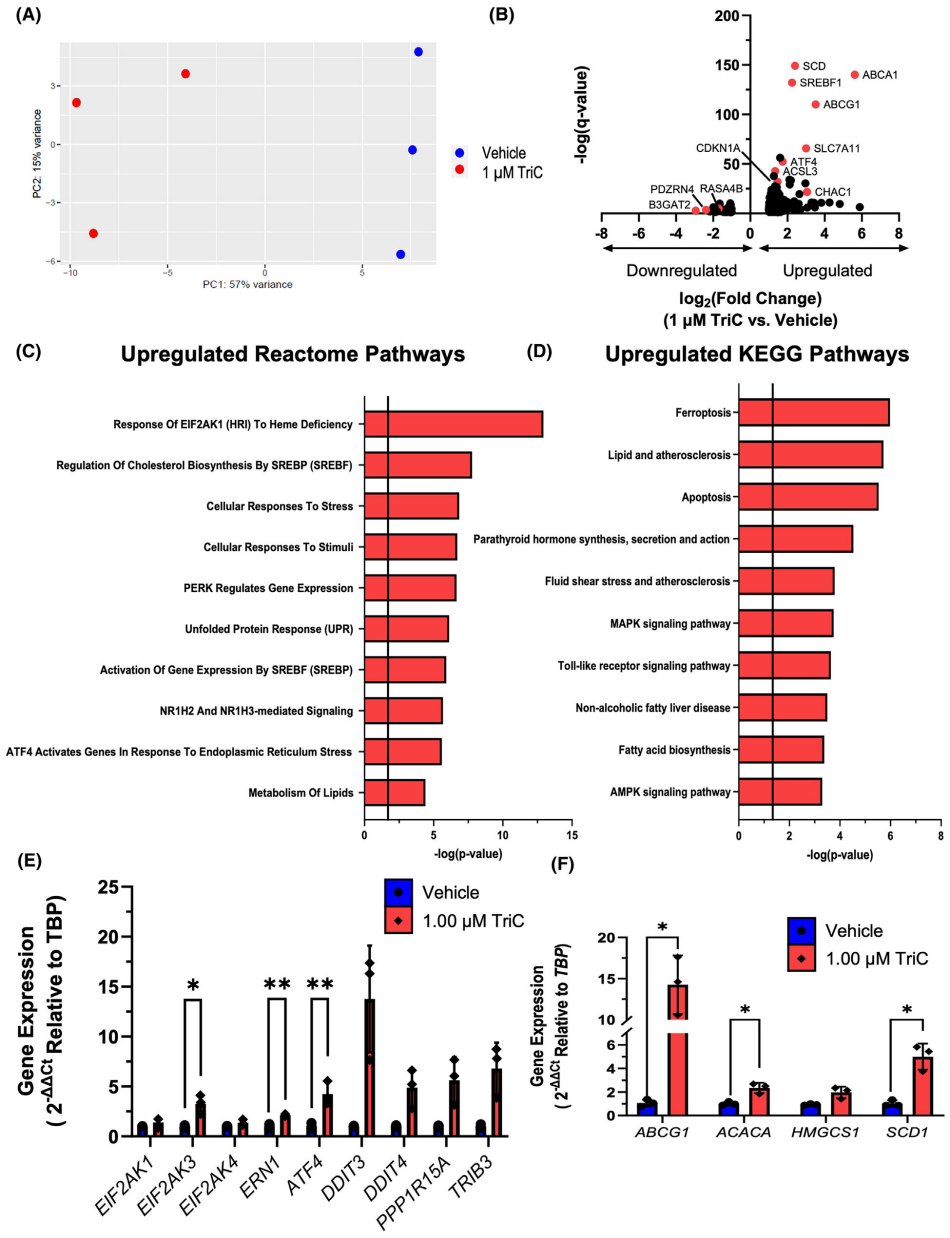
Triacsin C Treatment of MM.1S Cells Induces Transcriptional Changes Associated with Cell Death and the Integrated Stress Response. (A) Principal component analysis (PCA) of RNA‐seq data of MM.1S cells treated with vehicle or 1 μm TriC for 24 h. Dots represent biological replicates (*n* = 3 per group). (B) Differentially expressed transcripts derived from RNA‐seq of MM.1S cells treated with vehicle or 1 μm TriC. Genes of interest based on function are highlighted by red. Values calculated based on averages of three biological replicates per group. (C, D) Reactome and KEGG pathways enriched in the significantly upregulated transcripts in TriC‐ treated MM.1S cells. Vertical black line represents location of *P* = 0.05. (E, F) Expression of genes related to *ATF4* signaling and ER stress (E) and fatty acid metabolism (F) in MM.1S cells treated with vehicle or 1 μm TriC for 24 h; *n* = 3. Statistics: (E–H) Significance was tested with an unpaired Student's *t*‐test or Welch's *t*‐test. All data are mean ± SD, *P < 0.05, **P < 0.01.

To better understand the mechanisms of TriC's effects, RNA‐sequencing analysis was performed on MM.1S cells treated *in vitro* with 1 μm TriC or vehicle for 24 h. Transcripts were aligned to the *Homo sapiens* hg38 genome, which includes 12 772 protein‐coding genes, all of which passed the read mapping and quality standards (Fig. S9A,B). The transcriptomes of vehicle‐ and TriC‐treated cells were distinct by principal component analysis (PCA) and comparison of Euclidean distances (Fig. 2A; Fig. S9C) and 208 differentially expressed genes (DEGs; log_2_(FC) > |1|, *P*_adj_ < 0.05) were identified (167 upregulated and 41 downregulated, Fig. 2B). Reactome and KEGG pathways of upregulated DEGs found from RNA‐sequencing data included Cellular Response to Stress, ATF4 Activation in Response to ER Stress, Ferroptosis, and Apoptosis (Fig. 2C,D, Tables S4 and S5). Many of the downregulated Reactome and KEGG pathways related to proliferation and ECM/integrin signaling (Fig. S9D,E, Tables S6 and S7).

Next, to confirm RNA‐sequencing data, we performed qRT‐PCR. We first explored genes related to ATF signaling and ER stress. Indeed, we observed significantly increased gene expression of *EIF2AK3* (3.2 fold) and *ATF4* (4.2 fold) and a trend of increased expression in a number of downstream targets, such as *PPP1R15A* (5.6 fold), *TRIB3* (6.7 fold) and the pro‐apoptotic *DDIT3* (13.7 fold) in MM.1S cells treated with TriC (Fig. 2E). Expression of *ERN1*, another gene in the ER stress pathway, was also increased with TriC (Fig. 2E). There were also significant increases in genes involved in fatty acid metabolism, such as *ACACA* (2.3 fold) *SCD1* (5.0 fold), and the cholesterol transporter *ABCG1* (14.2 fold) (Fig. 2F).

Interestingly, expression of oncogene/metastasis‐associated genes *PAK6* and *RASA4B* decreased (Fig. S9F) and the cell cycle inhibitor *CDKN1A* increased (2.9 fold) (Fig. S9G). In addition, expression of both pro‐ferroptotic (*HMOX1*, *CHAC1*) and anti‐ferroptotic (*SLC3A2, SLC7A11*) genes increased (Fig. S9H). Thus, MM.1S cells treated with TriC have a transcriptional profile associated with ATF4 activation, apoptosis, and negative regulation of cell cycle progression.

### 3.4 Triacsin C treatment induces proteomic changes associated with mitochondrial dysfunction and reactive oxygen species detoxification

**Fig. 3:**
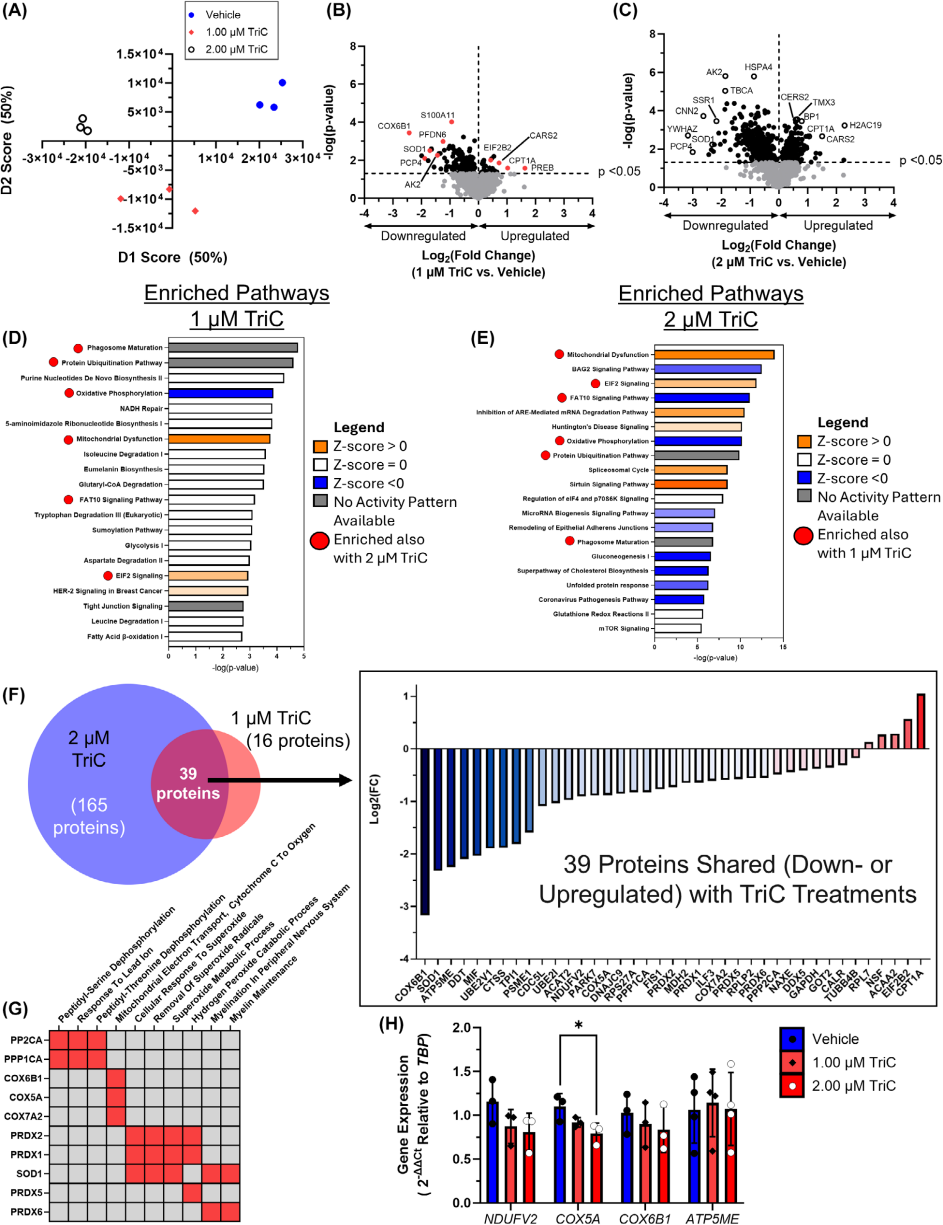
Triacsin C (TriC) Treatment of MM.1S Cells Induces Proteomic Changes Associated with Mitochondrial Dysfunction and Reactive Oxygen Species Metabolism. (A) Principal component analysis (PCA) of the proteomic profiles of MM.1S cells treated with Vehicle (blue circles), 1 μm TriC (red diamonds) or 2 μm TriC (open black circles); *n* = 3. (B, C) Volcano plots of differentially expressed proteins in MM.1S cells treated for 48 h with 1 or 2 μm TriC. Dashed lines represent *P* < 0.05 (horizontal) and log_2_(fold change) = 0 (vertical). Proteins of interest based on function are highlighted by red (B) or open circles (C); *n* = 3. (D, E) Top 20 enriched pathways in MM.1S cells treated with 1 and 2 μm TriC, respectively, identified using Ingenuity Pathway Analysis with their associated ‐log(*P*‐value). (F) DeepVenn depiction of the number of significantly changed proteins among the shared significantly changed pathways identified by Ingenuity Pathway Analysis (IPA) between 1 μm TriC (16 unique proteins) and 2 μm TriC (165 unique proteins) with a total of 39 shared proteins. The log_2_(fold change) is depicted for the 39 shared proteins among MM.1S cells treated with 1 or 2 μm TriC; *n* = 3. (G) Gene ontology (GO) enrichment for GO Biological Process of the 39 shared dysregulated proteins in MM.1S cells treated with 1 and 2 μm TriC for 48 h as assessed by Enrichr. A selection of proteins with common aberrant expression in the presence of both TriC doses are depicted here with red boxes indicating association between the protein and the GO term; gray boxes indicate no association. (H) Expression of genes related to oxidative phosphorylation in MM.1S cells treated with vehicle or TriC for 48 h, as assessed by qRT‐PCR; *n* = 3. Statistics: (H) Two‐way ANOVA with Šídák's multiple comparisons test and (B, C) Student's *t*‐test to identify differentially expressed proteins. All data are mean ± SD, *P < 0.05.

To identify cellular protein changes induced by ACSL inhibition, we treated MM.1S cells with 1 or 2 μm TriC, or vehicle for 48 h and analyzed them with sequential window acquisition of all theoretical fragment ion spectra (SWATH) mass spectrometry. The proteome of each group was well‐defined and functionally distinct from that of the others, as assessed by PCA (Fig. 3A). Of the approximately 1580 total proteins detected in TriC‐treated MM.1S cells, 167 and 614 differentially expressed proteins were detected in the 1 and 2 μm TriC‐treated cells, versus vehicle, respectively. The majority of differentially expressed proteins in both TriC treatments decreased (81.4% and 61.7%, 1 and 2 μm, respectively) relative to vehicle‐treated cells (Fig. 3B,C). Ingenuity Pathway Analysis (IPA) [52] revealed six shared pathways for MM.1S cells treated with either TriC dose: phagosome maturation, protein ubiquitination, FAT10 signaling, mitochondrial dysfunction, oxidative phosphorylation, and EIF2 signaling (Fig. 3D,E). Interestingly, mitochondrial function and EIF2 signaling were predicted to be activated, while oxidative phosphorylation was likely inactivated (*Z* score ≥ |2|) based on the differential protein expression of both the 1 and 2 μm TriC conditions compared to the vehicle (Fig. 3D,E). Within these six shared dysregulated pathways, 39 differentially expressed proteins were common to both TriC doses (Fig. 3F, Table S8), and these were significantly enriched for Biological Processes (identified using Enrichr) in two major categories: reactive oxygen species metabolism (SOD1, and PRDX1, 2, 5, and 6) and mitochondrial electron transport (COX6B1, COX5A and COX7A2) (Fig. 3G). Indeed, qRT‐PCR gene expression of *COX5A*, a subunit of Complex IV, was significantly decreased in MM.1S cells treated with TriC for 48 h, with similar but non‐significant trends for decreased expression of *COX6B1* and *ATP5ME* (Fig. 3H).

### 3.5 Triacsin C negatively impacts multiple myeloma cellular metabolism and mitochondrial function

**Fig. 4:**
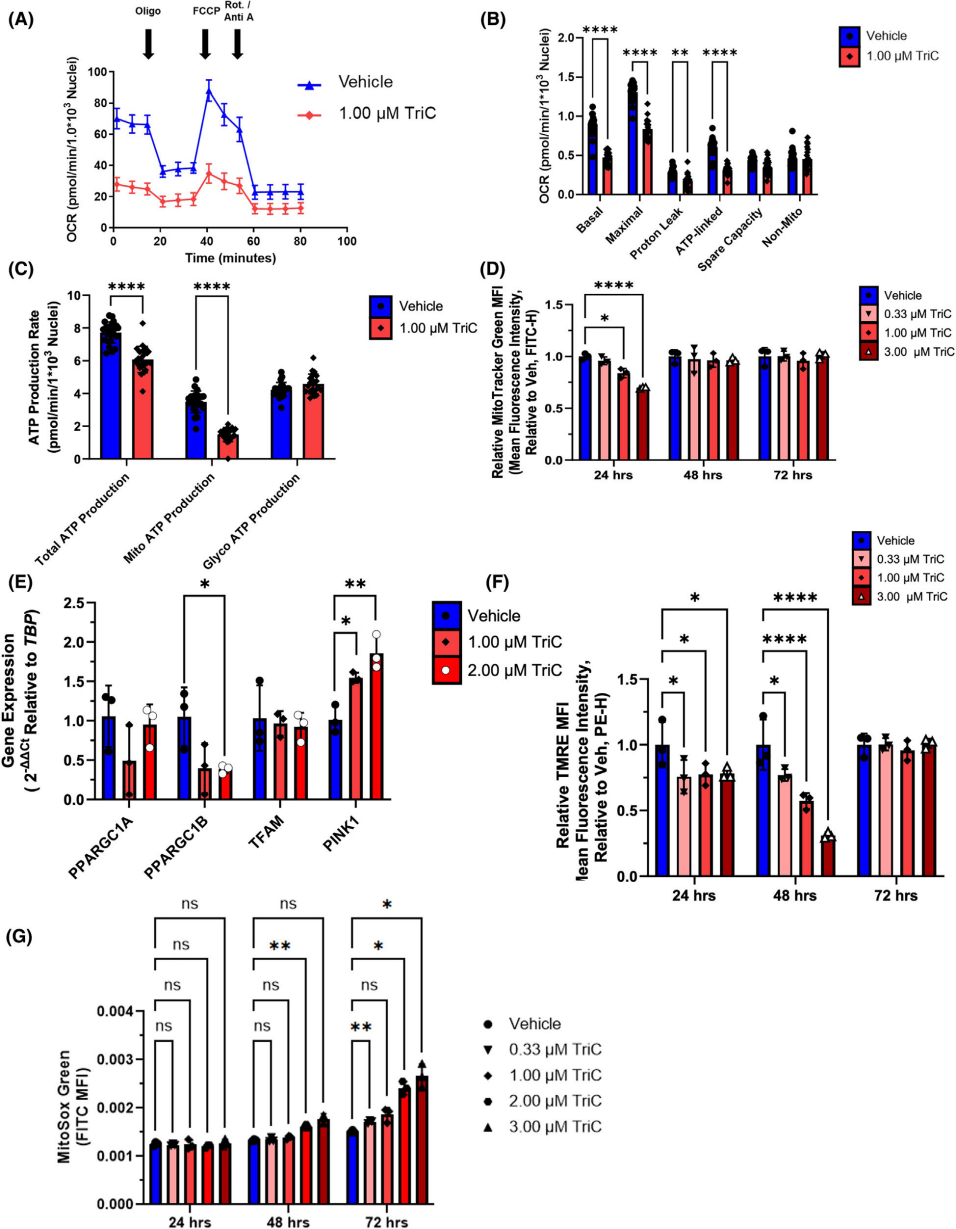
Triacsin C (TriC) Induces Mitochondrial Dysfunction and Rewires Mitochondrial Metabolism in Multiple Myeloma Cells. (A–C) Energy metabolism in MM.1S cells treated with 1 μm TriC for 24 h and subjected to SeahorseXFe96 Mito Stress test to measure oxygen consumption rate (OCR). Values are normalized to the number of nuclei. Data are representative of 3 independent experiments. Oligo = oligomycin (Complex V inhibitor), FCCP = (carbonyl cyanide p‐trifluoro methoxyphenylhydrazone, proton gradient uncoupler), Rot/Anti A = Rotenone/antimycin A (Complex I and III inhibitors, respectively). In C, Total, Glycolytic, and Mitochondrial ATP Production Rates in MM.1S cells treated with 1 μm TriC are shown. (D) Number/Mitochondrial Mass assessed with MitoTracker Green (100 nm) in MM.1S cells treated with TriC for 24, 48, and 72 h; *n* = 3. (E) Expression of genes related to mitochondrial biogenesis in MM.1S cells treated with vehicle, 1 or 2 μm TriC for 48 h; *n* = 3. (F) Assessment of mitochondrial integrity in MM.1S cells treated with TriC for 24, 48, and 72 h with TMRE staining; *n* = 3. (G) Mitochondrial superoxide quantification (MitoSox Green) in MM.1S cells treated with TriC for 24, 48, and 72 h; *n* = 3. Statistics: Two‐way ANOVA with Tukey's multiple comparison test used throughout except for (E), where a One‐way ANOVA with Dunnett's multiple comparisons test was used. Data are mean ± SD. *P < 0.05, **P < 0.01, ****P < 0.0001.

Given the mitochondria‐related findings from the RNA‐seq and proteomics analyses, we next tested the hypothesis that TriC functionally impairs myeloma cell metabolism and mitochondrial function. We treated MM.1S cells with 1 μm TriC for 24 h and then subjected them to mitochondrial stress measurements (Fig. 4A). TriC‐treated cells had significantly reduced basal, maximal, and ATP‐dependent mitochondrial respiration and proton leakage (Fig. 4A,B). Interestingly, in parallel samples, we observed a significant 21.27% decrease in total ATP production rates attributable to a 57.3% decrease in mitochondrial ATP production rates, with no significant compensatory increase in glycolytic ATP production rates in TriC‐treated cells (Fig. 4C). Taken together, these data demonstrate that ACSL inhibition reduces mitochondrial ATP production rates and cellular respiration in myeloma cells.

Next, to determine TriC effects on mitochondrial biogenesis, total mitochondria were quantified (Fig. 4D). TriC‐treated MM.1S cells exhibited a dose‐dependent decrease in the relative mean fluorescence intensity (MFI) of MitoTracker Green after 24 h, but not at 48 or 72 h, perhaps due to a recovery or compensation response. MM.1S cells treated with 1 or 2 μm TriC after 48 h showed a decrease in expression of a key mitochondrial biogenesis gene, *PPARGC1B*, and an increase in a mitochondrial quality control gene activated by cellular stress, *PINK1* (Fig. 4E). We next tested the mitochondrial membrane potential effects of TriC in MM.1S cells using tetramethylrhodamine ethyl ester (TMRE). Consistent with our prior data supporting mitochondrial dysfunction, we observed a dose‐dependent decrease in the TMRE signal (mitochondrial membrane potential) in cells treated with TriC after 24 and 48 h (Fig. 4F). Since mitochondrial dysfunction is often associated with an increase in oxidative stress and superoxide formation, we predicted that TriC‐treated MM cells would exhibit increased ROS and lipid peroxidation. Interestingly, MM.1S cells treated with TriC showed time‐ and dose‐dependent increases in mitochondrial superoxide levels based on MitoSOX Green staining (Fig. 4G). Consistent with increased superoxide levels, we also observed a decrease in RealTime‐Glo MT signal in MM.1S and OPM2 cells over time with TriC treatment, in parallel to an increase in peroxidation of their lipids using a ratiometric BODIPY 581/591 assay (Fig. S10). Taken together, ACSL‐inhibited myeloma cells had decreased oxygen consumption, mitochondria‐derived ATP production rates, total mitochondrial mass, mitochondrial membrane potential, and cell viability, along with increased superoxide and lipid peroxidation levels. Overall, these data support the hypothesis that targeting ACSL proteins in myeloma represents a novel metabolic target for inducing oxidative stress and restraining myeloma tumor growth that would benefit from further investigation.

## 4 Discussion

In the present study, we identified the ACSL family of ligases as clinically relevant and supportive of myeloma cells. ACSL1, ACSL3, ACSL4, and ACSL5 are expressed in primary myeloma cells and supportive of myeloma cell fitness, suggesting that broad targeting of the ACSLs could be impactful for MM patients. Pharmacological inhibition of the ACSL family with TriC decreased human myeloma cell viability and proliferation, and induced apoptosis and mitochondrial dysfunction. The viability EC_50_ of TriC in myeloma cells ranged from 1.44 to 8.56 μm at 48 h, which is similar to values reported for TriC *in vitro* in many other cell lines (breast cancer (2.4–7.2 μm depending on cell line) [20], Burkitt's lymphoma (~ 1 μm) [51], and lung, colon, and brain cancer cell lines (~ 3–9 μm) [53]). Importantly, work from Mashima et al. [53] found that non‐cancerous cell lines (with normal p53 status) are less sensitive to TriC than cancer cell lines with p53 mutations, suggesting a potential for safe use of ACSL‐targeting therapies. Interestingly, in our hands, while most of the MM cell lines tested were *TP53* mutants, MM.1S (*TP53* ^WT^) cells were sensitive to TriC treatment, suggesting that TriC toxicity in myeloma cells may be independent of *TP53* status. Importantly, TriC showed no toxicity to healthy donor CD34^+^ cord blood cells in the dose range tested (up to 16 μm, 48 h) [21] or in primary rat hepatocytes with treatments of up to 5 μm for 8 h [54]. Similarly, primary mouse cardiomyocytes had no viability effects after a 6 h 10 μm TriC treatment *in vitro* [55], and TriC slowed lung cancer growth without causing any toxicity to nude mice [21]. This provides further support for the potential to use ACSL‐targeting therapies at effective doses without unacceptable toxicity. To address the possibility of TriC‐dependent off‐target effects, investigate compensation mechanisms between ACSL proteins, and dissect specific biological roles for each ACSL member, future studies using genetic (knockdown or knockout) approaches to inhibit ACSL isozymes individually in myeloma cells are warranted.

The major hurdle in moving this research to humans is that drugs targeting the ACSLs are not clinically available. Triascin C itself is not a useful pharmacological agent due to its pharmacokinetic and cell penetrance properties (as reviewed here [56]), and despite many efforts, no compound has been developed with a higher inhibition rate of the ACSLs than Triacsin C. Moreover, ACSL inhibitors are not specific since the five ACSL isoenzymes have a conserved catalytic domain [56]. Perhaps newer technologies will address this challenge using drug optimization (e.g., liposomes, drug‐antibody conjugates) or tumor‐specific delivery techniques. Overall, drug development will be required before pharmacological targeting of ACSLs clinically can be pursued.

There is potential for synergy between anti‐ACSL and anti‐myeloma therapies, as we observed that the dexamethasone‐resistant MM.1R cell line was sensitive to TriC. Moreover, the CoMMpass dataset analysis showed that low expression of *ACSL4* correlated with not only better overall survival, but also increased time to second‐line treatments for all patients, and for those treated only with the combined bortezomib/IMiDs‐based first‐line therapy. However, this was not seen with other ACSL family members: high *ACSL1* expression only correlated with worse overall survival and not worse time to second‐line treatment, suggesting targeting ACSL4 and ACSL1 may have slightly different effects and thus, potentially additive value. TriC has also shown additive or synergistic effects with other therapies (e.g., increasing sensitivity to gemcitabine in pancreatic and lung cancer cells [57, 58] and synergizing with ABT‐199, the FDA‐approved BCL‐2 inhibitor, in AML cells [21]). Similarly, endometrial cell lines were sensitized to TriC by the addition of omega‐3 FA docosahexaenoic acid (DHA), suggesting that combinations of TriC with other metabolic effectors or therapies may increase the relevance of TriC or ACSL‐targeting therapies in myeloma [23]. TriC can also synergize with etoposide [22] and a combination of anti‐metabolites and alkylating agents in glioma and colorectal cancer [59]. Lastly, TriC has been shown to reduce tumor cell lipid droplets [60], which may contribute to drug resistance in CRC [59]. Additionally, inhibition of ACSLs may increase tumor cell sensitivity to venetoclax, a BCL2 inhibitor [61], since electron transport chain Complex I and II activity is positively correlated with resistance to venetoclax, and we observed that TriC decreased expression of subunits of Complexes I and IV. Moreover, in AML, AMPK‐PERK‐ATF4 activation also confers sensitivity to venetoclax by repressing oxidative phosphorylation [62]. Therefore, future studies should test whether ACSL inhibition synergizes with venetoclax and explore targeting the ACSLs in combination with existing treatments for MM. We also suggest that primary patient tumor cells be explored for their response to ACSL inhibition in future work.

Distinct mechanisms of action have been implicated in the effects of TriC in other cells, including the reduction of key survival pathways (p38/MAPK [63], nuclear factor‐κB (NF‐kB) [63]) [ and increased BAX‐induced caspase activation [22]. In our work, myeloma cells exhibited a robust transcriptional and metabolic response to TriC, consistent with the observed decreased viability, proliferation, and metabolism, and increased apoptosis. Indeed, many pro‐apoptotic genes downstream of the ATF4‐eIF2S1 pathway [64], such as *DDIT3* and *TRIB3*, showed a trend for increased expression upon TriC treatment. Two upstream activators of the ATF4‐eIF2S1 pathway (PERK/EIF2AK3 and HRI/EIF2AK1) were enriched pathways and activation of ATF4 via these pathways has been shown to induce apoptosis in AML [62] and MM [65, 66]. These data suggest that apoptosis may be initiated in an ATF4‐eIF2S1‐dependent manner and should be addressed in future studies.

Myeloma cellular responses to ACSL inhibition were interconnected and did not allow for a straightforward picture of how cells death occurred. TriC treatment reduced rates of ATP generation from the mitochondria and it is unclear if this was due to reduced mitochondria or compromised mitochondrial function, since both were observed. Superoxides and lipid peroxidation (the oxidative degradation of cellular lipids by free radicals from ROS) were also increased by TriC, which can affect metabolism, damage cell membranes, affect signal transduction pathways, and lead to cell death. Additionally, ferroptosis was the most upregulated KEGG pathways in MM cells treated with TriC, as assessed by RNA‐Seq, but we have not yet been able to confirm TriC‐induced ferroptosis in myeloma cells. We propose that future directions should test the specific role of ferroptosis in TriC‐induced tumor cell death. Additionally, the “Metabolism of Lipids” Reactome pathway was enriched in the upregulated RNA‐seq data. The majority of our lipid metabolism‐centered gene expression data related to anabolic processes, suggesting that myeloma cells may have been responding to lipid starvation, which is consistent with the predicted outcome of ACSL inhibition.

## 5 Conclusions

In summary, our data support the hypothesis that ACSLs maintain myeloma cell fitness and viability though effects on cellular metabolism, mitochondrial function, survival pathways, proliferation rates, stress responses, and superoxide levels. ACSLs represent promising therapeutic targets for MM and further research and development is needed to translate this preclinically identified target into clinical benefit. Overall, our work demonstrates the importance of cell intrinsic fatty acid metabolism in oncology and demonstrates the potential for novel therapeutics, or other interventions, to interfere in this metabolic pathway within tumor cells.

## Conflict of interest

Habib Hamidi and Xiangnan Guan are employees and stockholders of Roche/Genentech. All other authors have no COIs.

## Author contributions

CSM: Conceptualization, resources, data curation, software, formal analysis, supervision, validation, investigation, visualization, methodology, writing‐original draft, project administration, funding acquisition, writing‐review, and editing. HF: Investigation, project administration, funding acquisition, writing, reviewing, and editing. VED: Resources, data curation, formal analysis, writing‐ original draft, writing–review, and editing. SF: Investigation. CAG: LC–MS data acquisition, data curation, investigation, methodology, writing, review, and editing. MK: Data curation. Reviewing. CP: Data curation, reviewing and editing. PR: Data curation, investigation, writing, reviewing, and editing. YWQ: Investigation, writing‐review, and editing. HH: Resources, data curation, software, formal analysis, writing‐editing. XG: software, formal analysis, writing‐editing. CPHV: curation, LC–MS data analysis, investigation, methodology, writing, reviewing, and editing. MRR: Conceptualization, resources, data curation, formal analysis, supervision, funding acquisition, validation, investigation, visualization, methodology, writing – original and final draft, project administration, writing – review and editing.

## Peer review

The peer review history for this article is available at https://www.webofscience.com/api/gateway/wos/peer‐review/10.1002/1878‐0261.13794.

## Supporting information


**Fig. S1.** MMRF bulk RNA‐sequencing data of CD138+ myeloma cells sorted from patient BM samples at baseline from the CoMMpass trial.
**Fig. S2.** High *ACSL1* expression in tumor cells correlates with worse overall survival for MM patients.
**Fig. S3.** Cox regression analysis of bulk RNA‐seq data of *ACSL4* (ENSG00000068366) expression in CD138‐positive cells from CoMMpass dataset.
**Fig. S4.** Cox regression analysis of scRNA‐seq data of all ACSL family members, from CD138‐negative cells in MM patient BM from CoMMpass dataset.
**Fig. S5.** Further Characterization of ACSLs in MM and Effects of TriC.
**Fig. S6.** Ki‐67 and Cell Cycle Example Analyses.
**Fig. S7.** Apoptosis and BAX Expression Example Analyses.
**Fig. S8.** Effects of TriC on human PBMCs.
**Fig. S9.** Supportive Data on RNA‐sequencing of MM.1S Cells Treated with TriC or Vehicle for 24 h.
**Fig. S10.** Triacsin C induces lipid peroxidation in myeloma cells while simultaneously decreased cell viability.
**Table S1.** Average Chronos Scores of Modified Hallmark Fatty Acid Metabolism Genes in 21 Human Myeloma Cell Lines from the Cancer Dependency Map version 22Q2.
**Table S2.** qRT‐PCR Forward Primers.
**Table S3.** qRT‐PCR Reverse Primers.
**Table S4.** Top 10 Significantly Upregulated Reactome Pathways in MM.1S Cells Treated with TriC for 24 h based on RNA‐sequencing data.
**Table S5.** Top 10 Significantly Upregulated KEGG Pathways in MM.1S Cells Treated with TriC for 24 h based on RNA‐sequencing data.
**Table S6.** Top 10 Significantly Downregulated Reactome Pathways in MM.1S Cells Treated with TriC for 24 h.
**Table S7.** All Significantly Downregulated KEGG Pathways in MM.1S Cells Treated with TriC for 24 h.
**Table S8.** Significantly Changed Proteins Shared Among Overrepresented Pathways between MM.1S cells Treated with 1 or 2 μm TriC for 48 h.

## Data Availability

Raw and normalized RNA‐Seq data are available from the Gene Expression Omnibus database (GSE252929). Mass spectrometry data are available in the PRIDE database, PXD049304.
