# Supplementary material for: Inhibition of acyl‐CoA synthetase long‐chain isozymes decreases multiple myeloma cell proliferation and causes mitochondrial dysfunction
Source: Mol Oncol. 2025 Jan 23;19(6):1687–706. doi: 10.1002/1878-0261.13794 (PMC12161464; doi:10.1002/1878-0261.13794)

Supp Figure 1

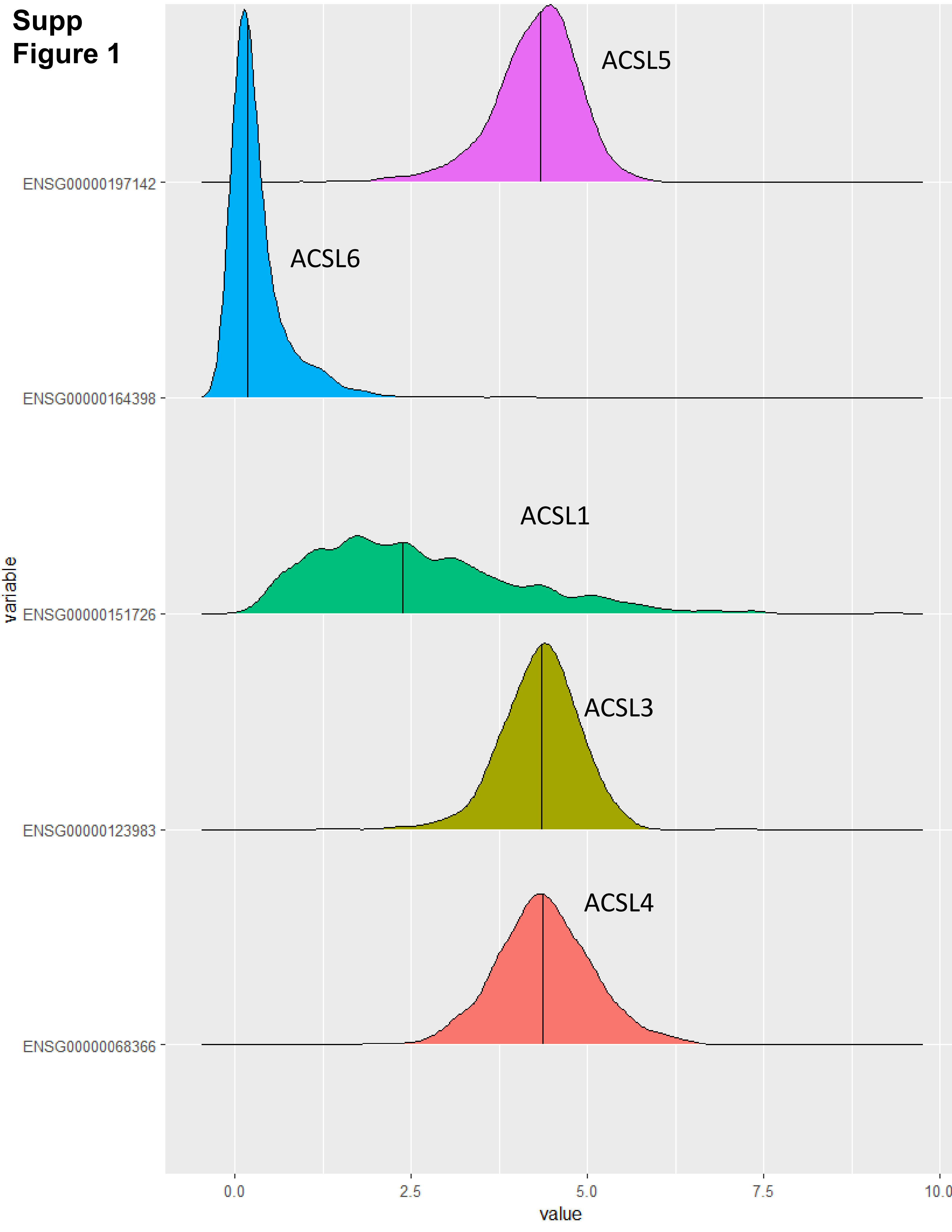

Supp Figure 2

ACSL1 Expression Hazard Ratio Table

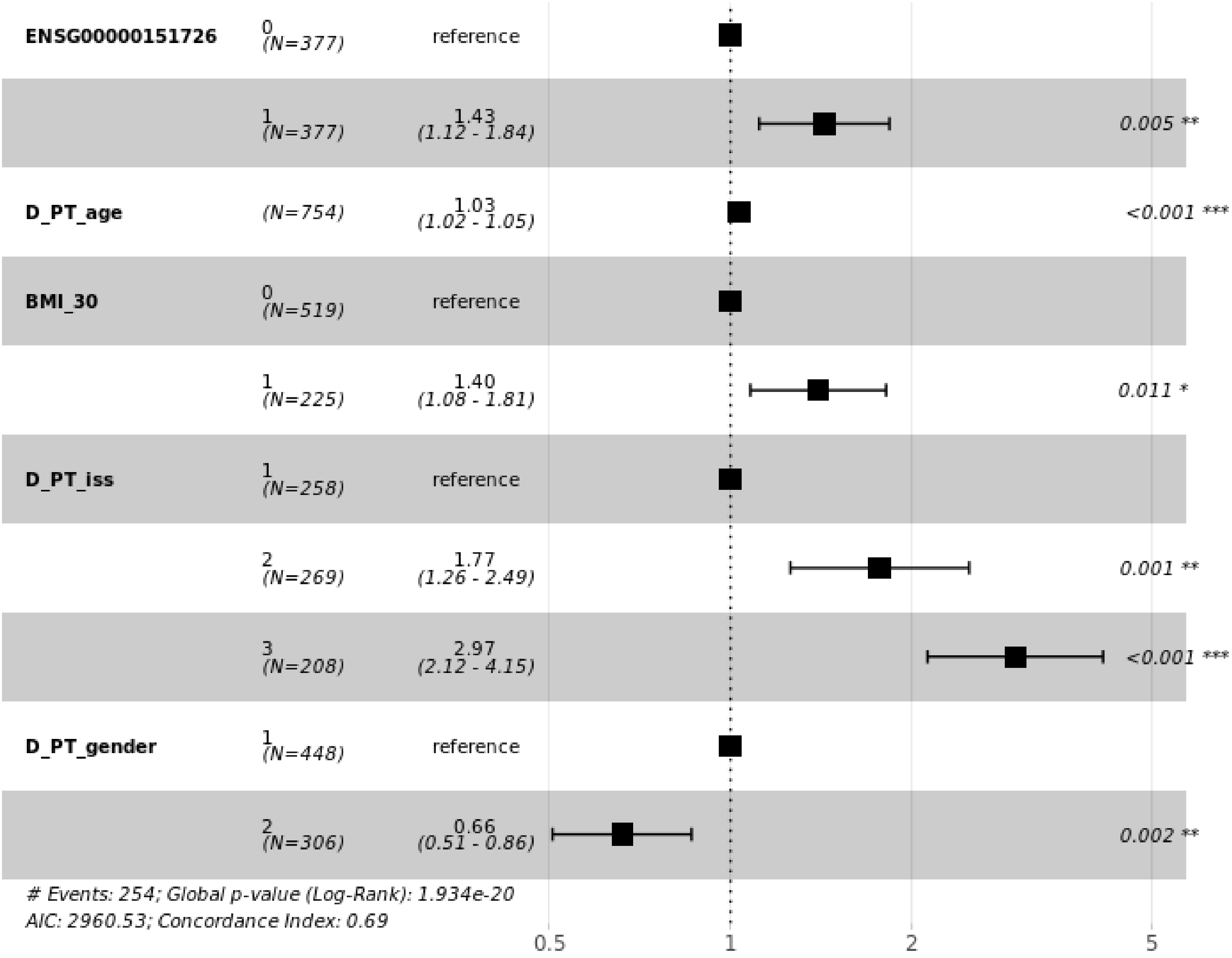

Supp Figure 3

A Overall Survival

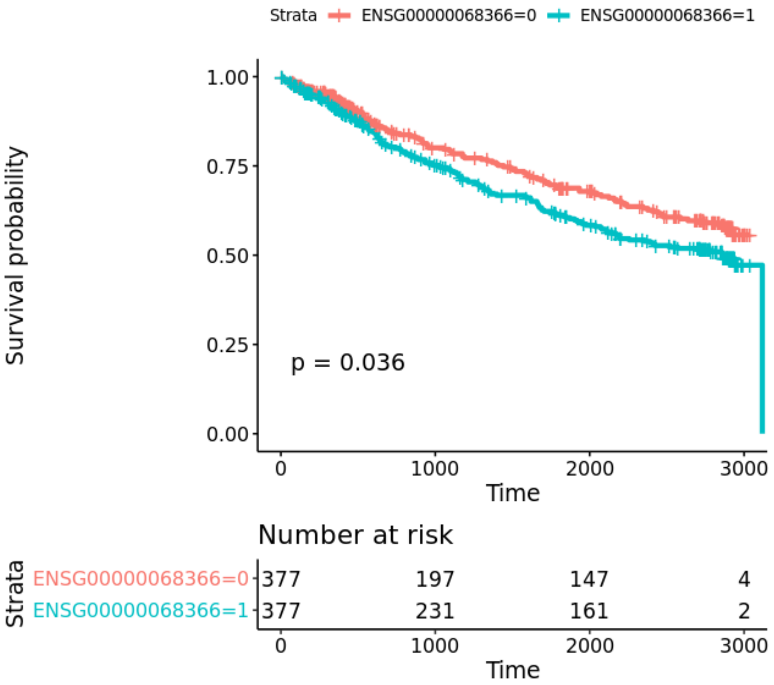

B Time to second line

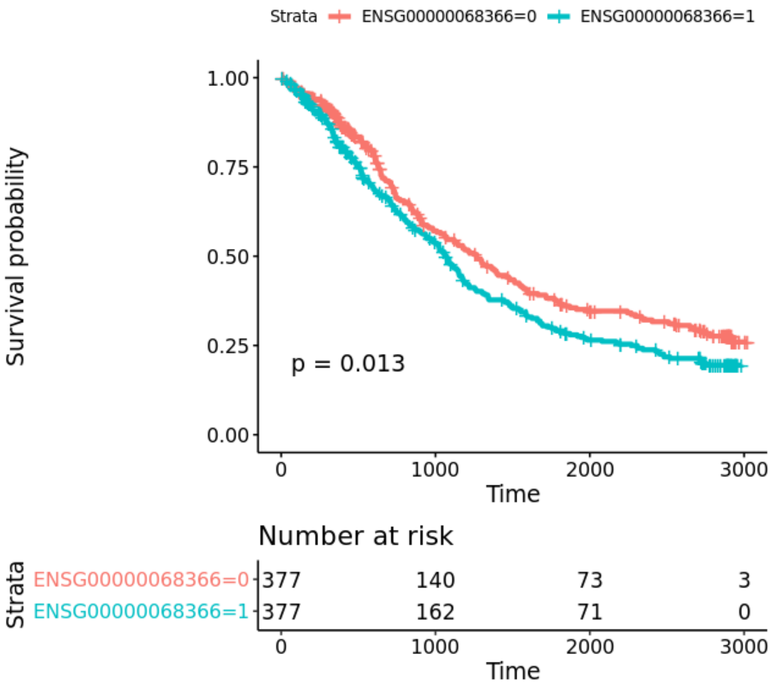

C Time to second line (patient subgroup)

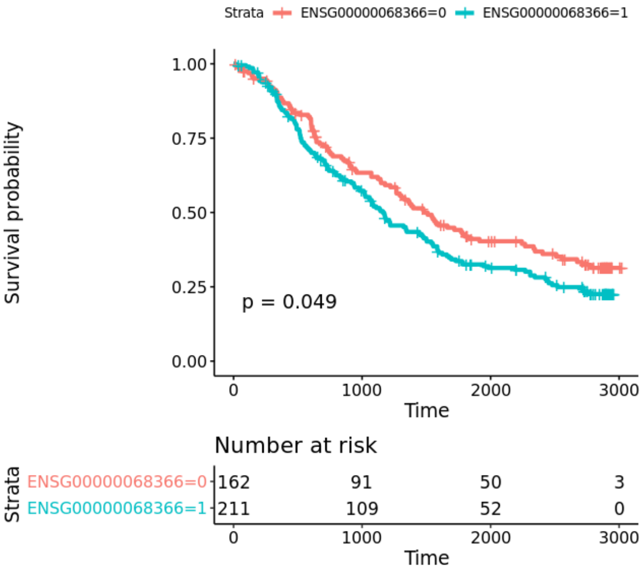

D Multivariate analysis using cox regression for Overall Survival

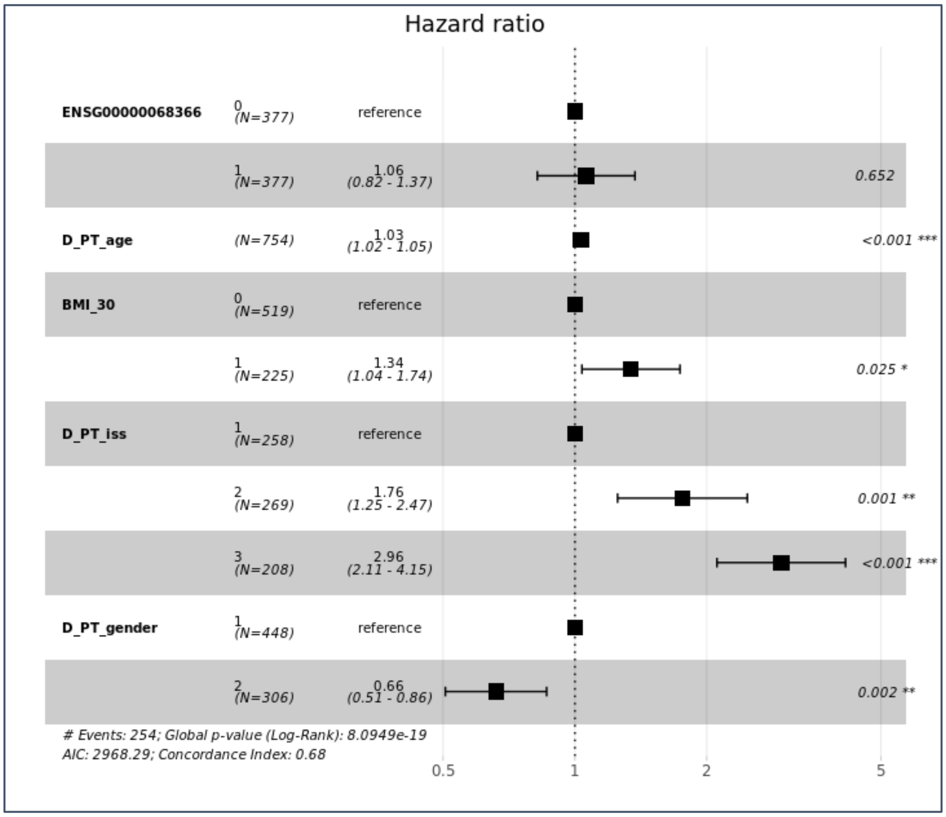

Supp Figure 4

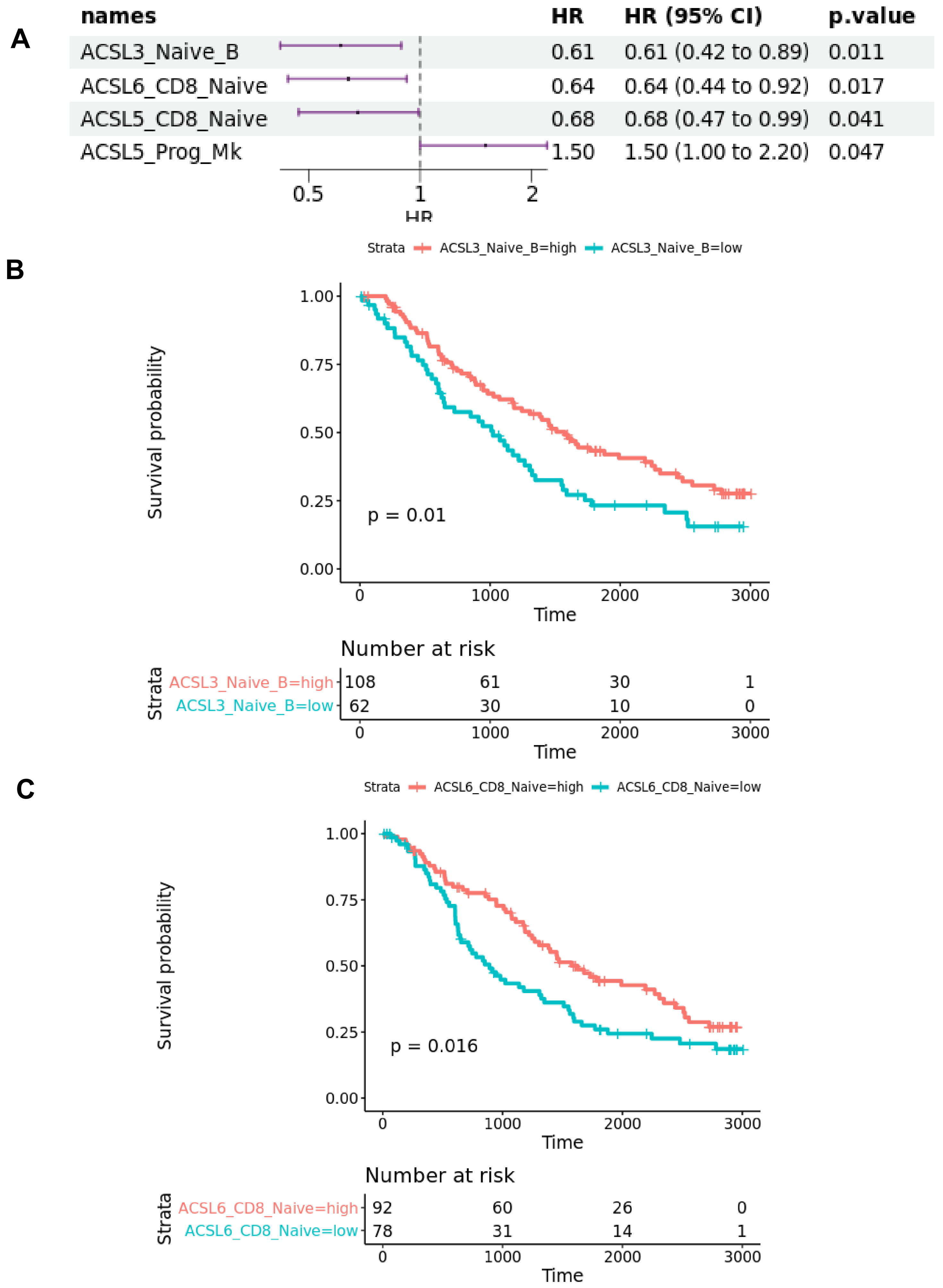

Supp Figure 5

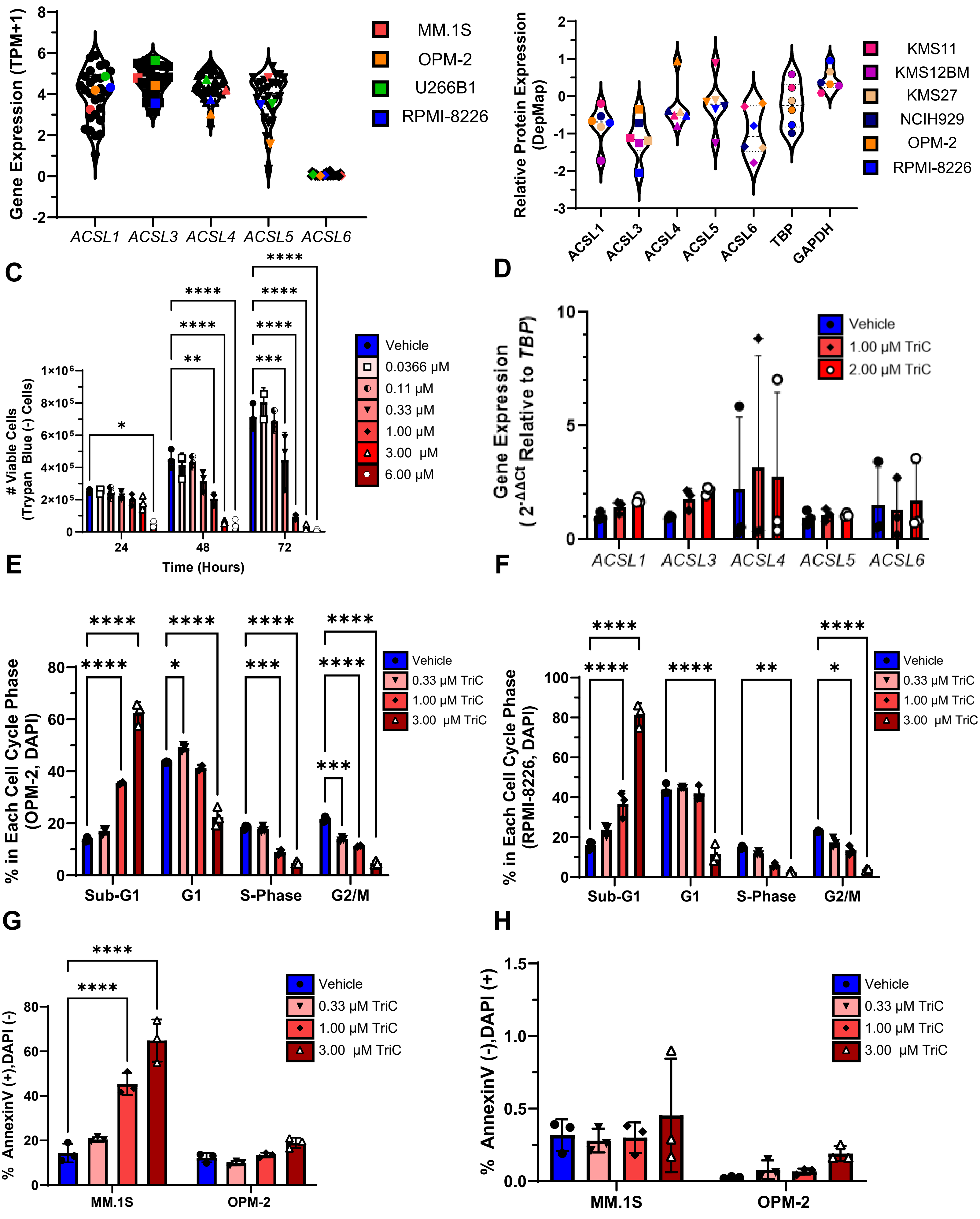

Supp Figure 6

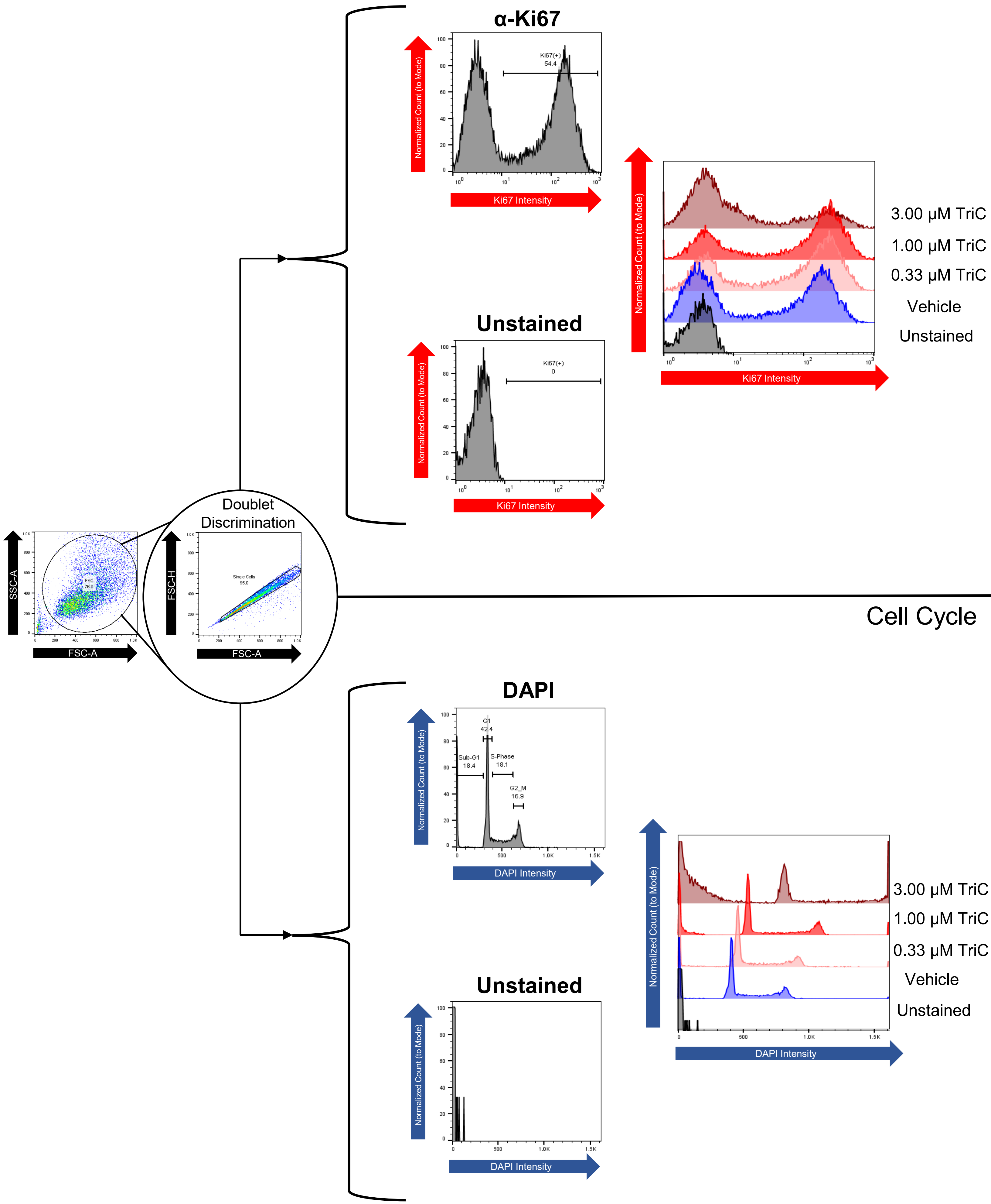

Supp Figure 7

A

Annexin-V and DAPI

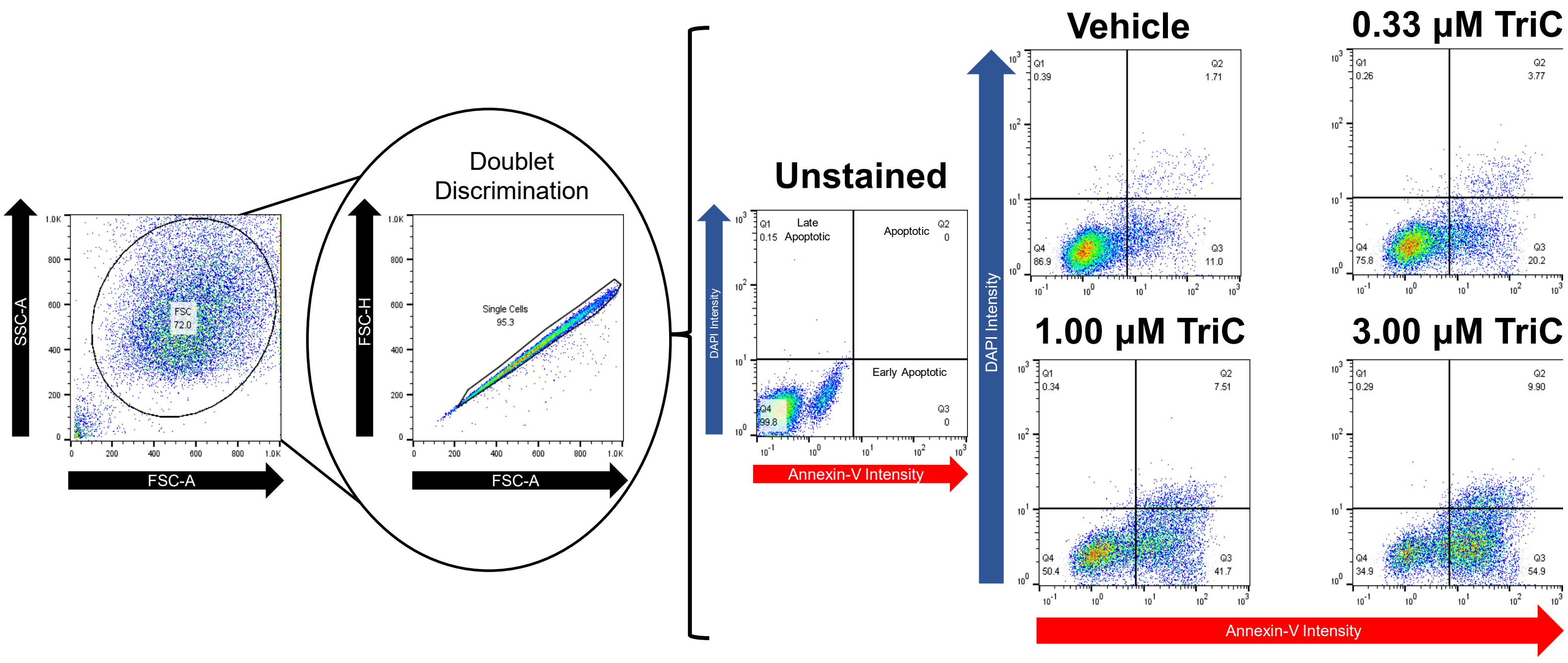

B

BAX

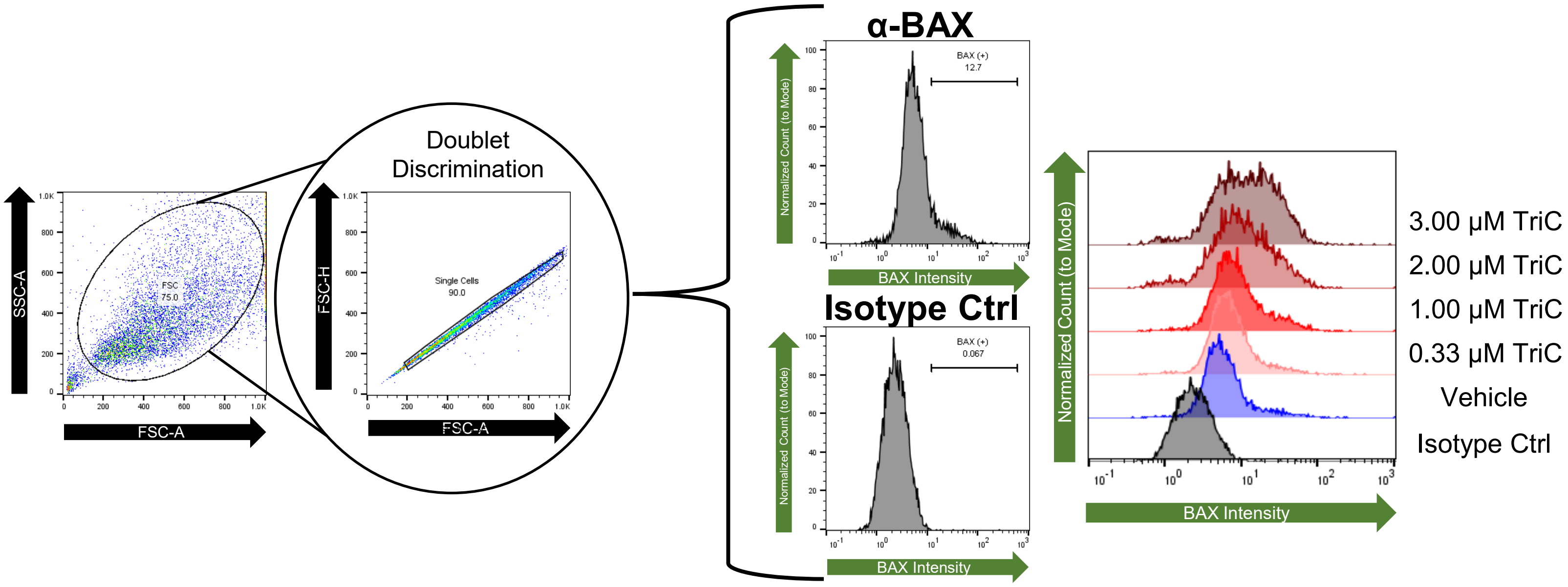

Supp Figure 8

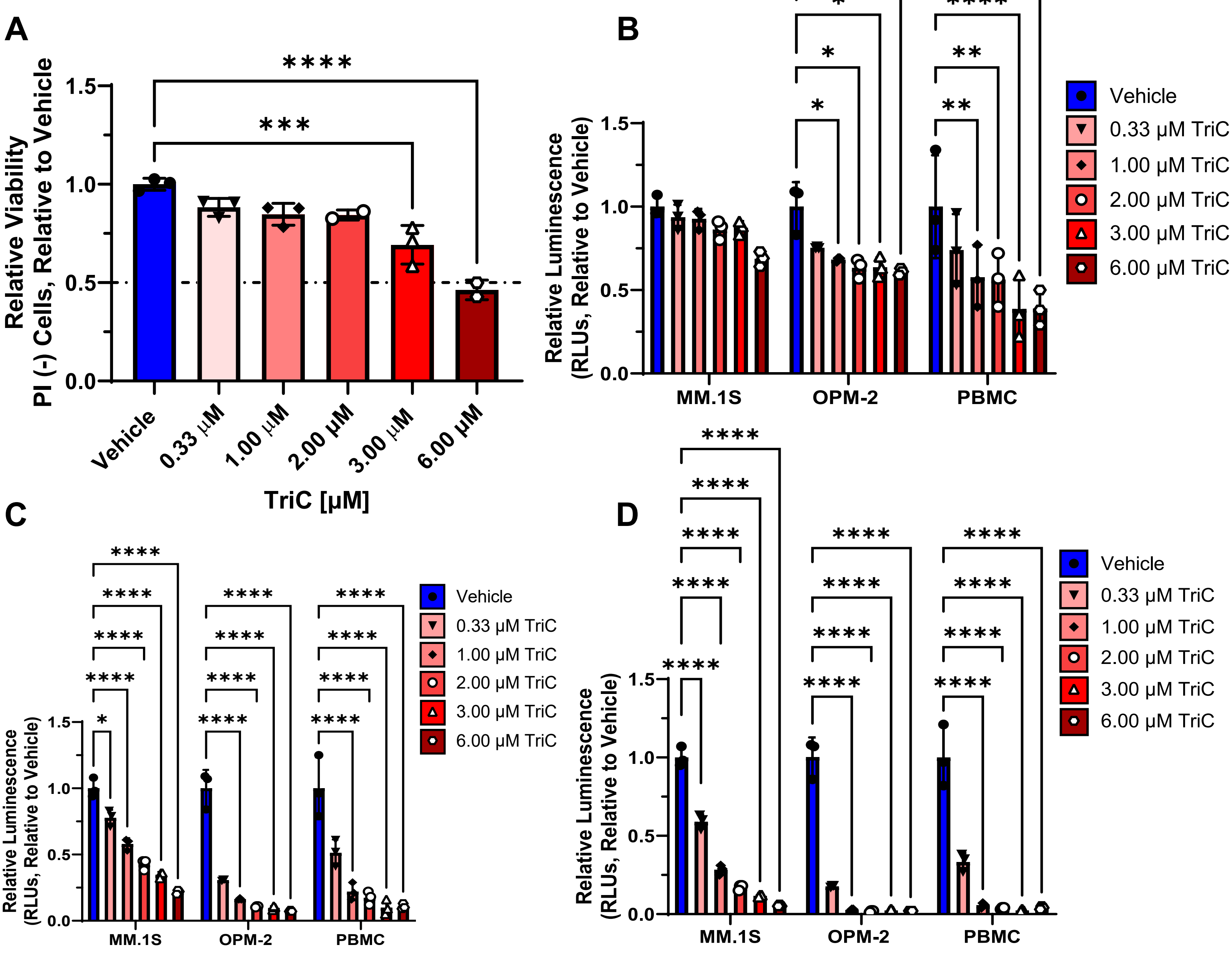

Supp Figure 9

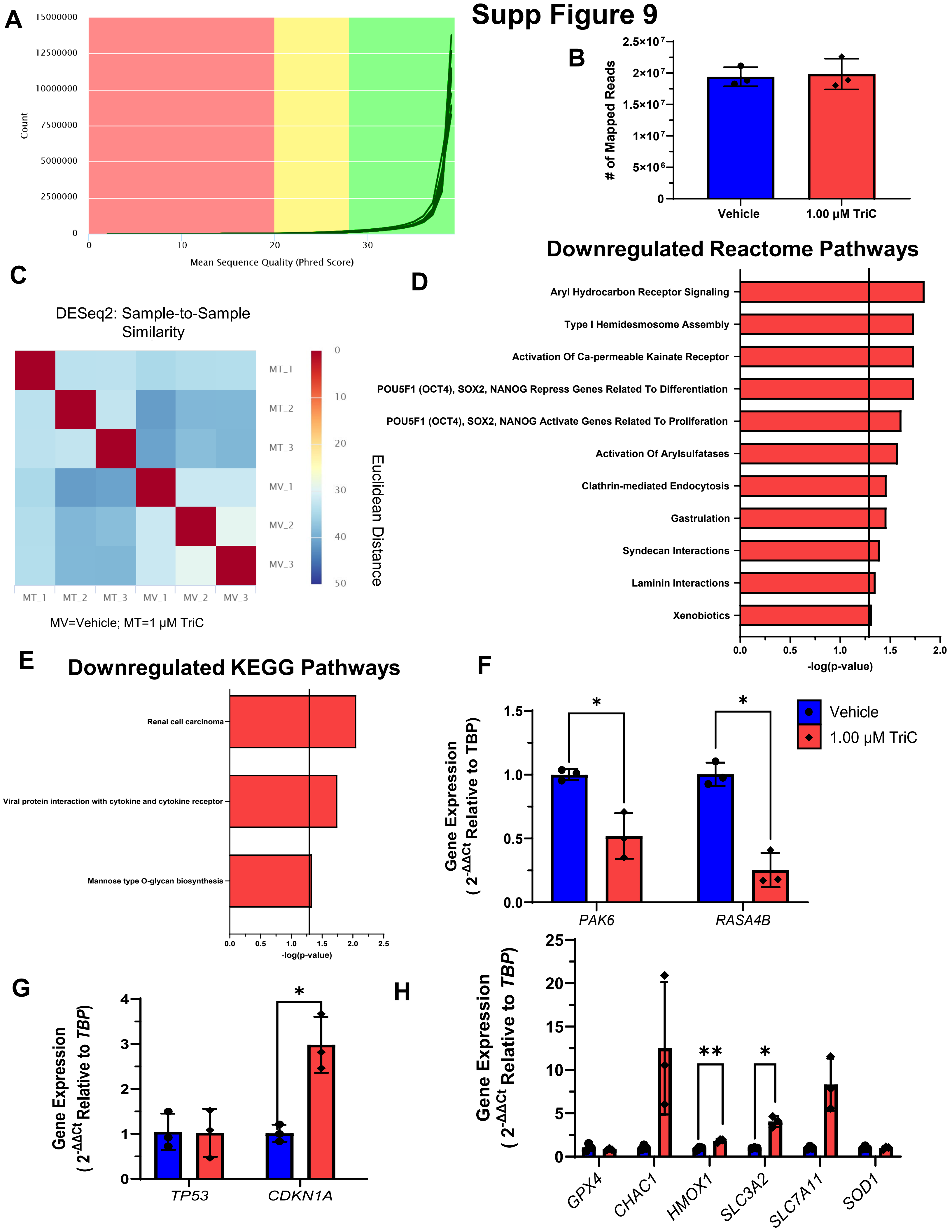

Supp Figure 10

A

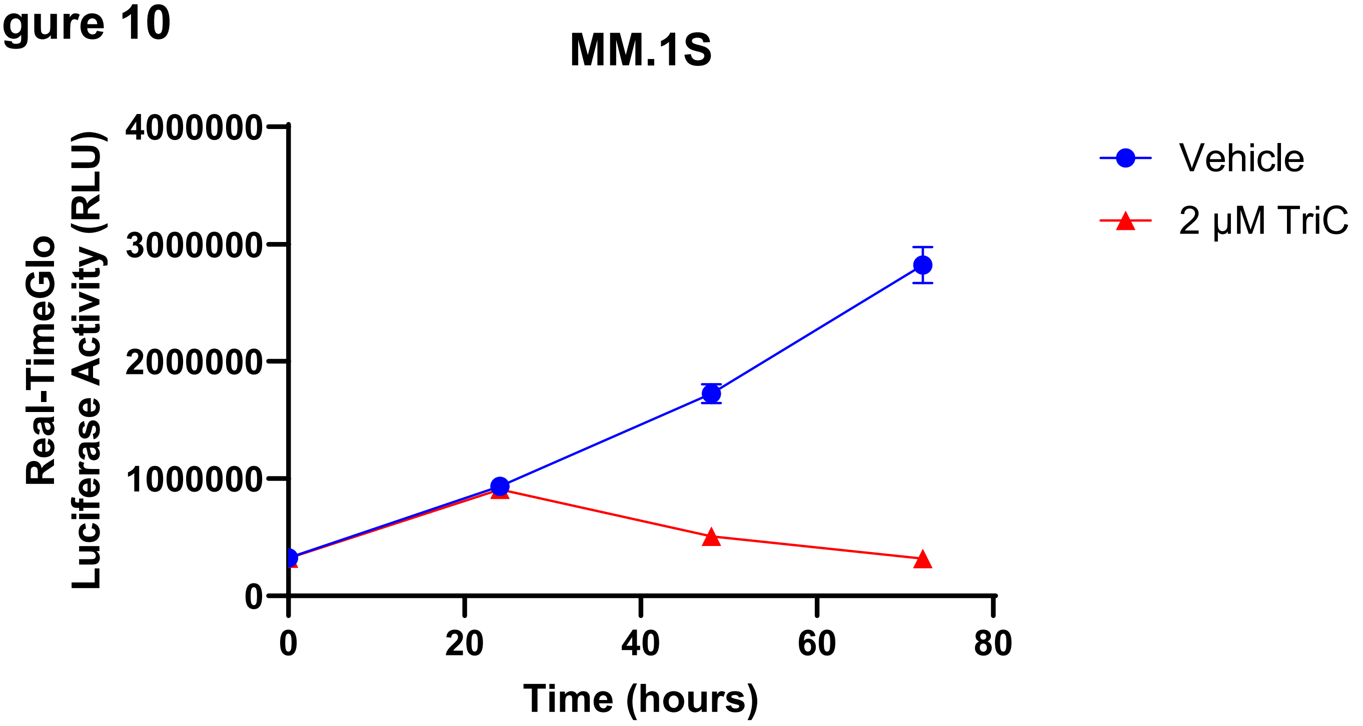

B

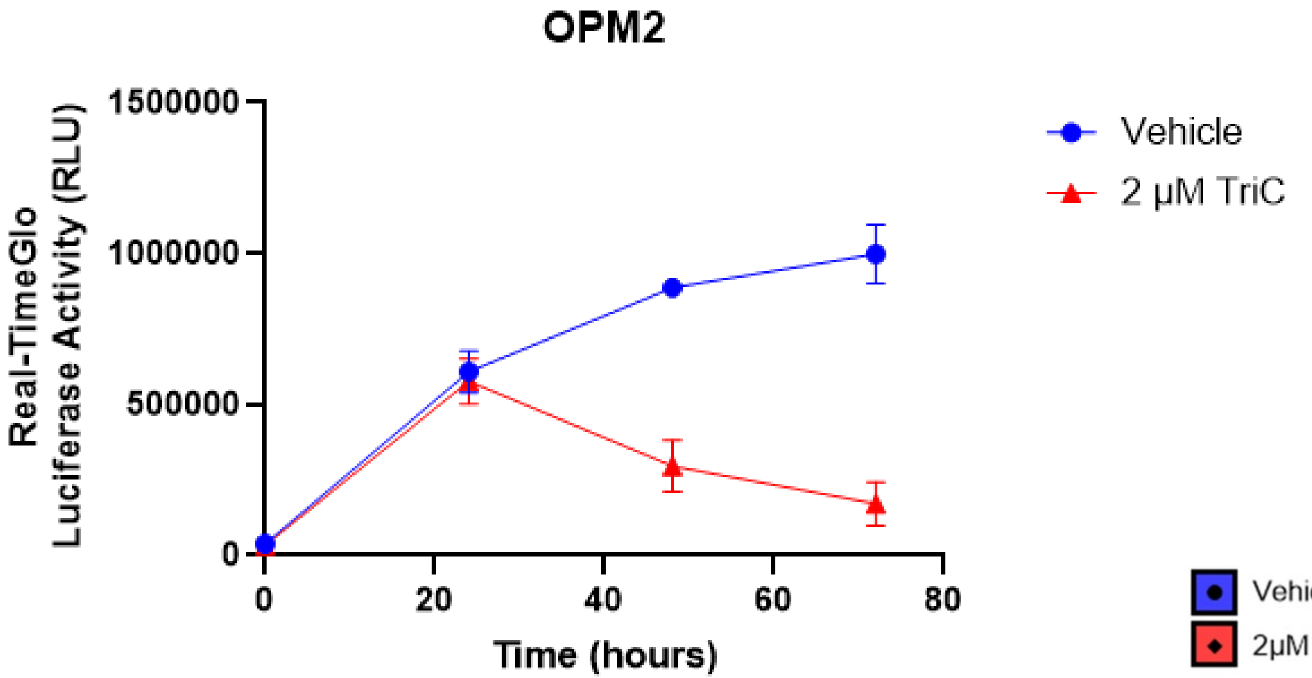

C

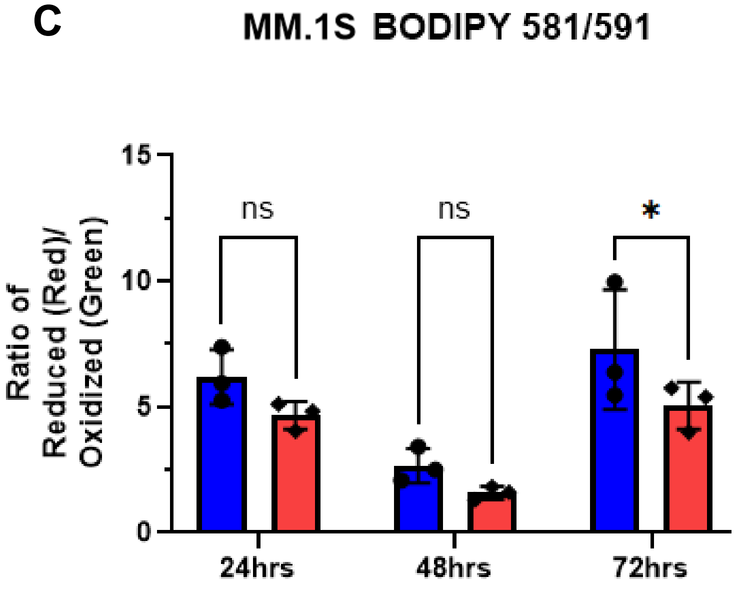

D

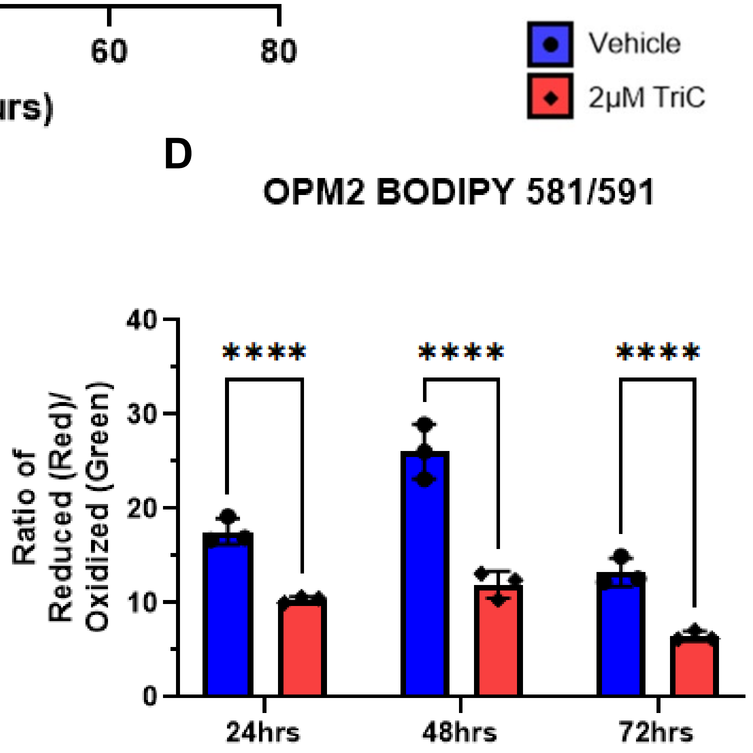

Supplement: Supplementary file 1 — Fig. S1. MMRF bulk RNA‐sequencing data of CD138+ myeloma cells sorted from patient BM samples at baseline from the CoMMpass trial. Fig. S2. High ACSL1 expression in tumor cells correlates with worse overall survival for MM patients. Fig. S3. Cox regression analysis of bulk RNA‐seq data of ACSL4 (ENSG00000068366) expression in CD138‐positive cells from CoMMpass dataset. Fig. S4. Cox regression analysis of scRNA‐seq data of all ACSL family members, from CD138‐negative cells in MM patient BM from CoMMpass dataset. Fig. S5. Further Characterization of ACSLs in MM and Effects of TriC. Fig. S6. Ki‐67 and Cell Cycle Example Analyses. Fig. S7. Apoptosis and BAX Expression Example Analyses. Fig. S8. Effects of TriC on human PBMCs. Fig. S9. Supportive Data on RNA‐sequencing of MM.1S Cells Treated with TriC or Vehicle for 24 h. Fig. S10. Triacsin C induces lipid peroxidation in myeloma cells while simultaneously decreased cell viability. Table S1. Average Chronos Scores of Modified Hallmark Fatty Acid Metabolism Genes in 21 Human Myeloma Cell Lines from the Cancer Dependency Map version 22Q2. Table S2. qRT‐PCR Forward Primers. Table S3. qRT‐PCR Reverse Primers. Table S4. Top 10 Significantly Upregulated Reactome Pathways in MM.1S Cells Treated with TriC for 24 h based on RNA‐sequencing data. Table S5. Top 10 Significantly Upregulated KEGG Pathways in MM.1S Cells Treated with TriC for 24 h based on RNA‐sequencing data. Table S6. Top 10 Significantly Downregulated Reactome Pathways in MM.1S Cells Treated with TriC for 24 h. Table S7. All Significantly Downregulated KEGG Pathways in MM.1S Cells Treated with TriC for 24 h. Table S8. Significantly Changed Proteins Shared Among Overrepresented Pathways between MM.1S cells Treated with 1 or 2 μm TriC for 48 h. [file MOL2-19-1687-s001.zip › Supp Figures for PDF.pdf]
