# Supplementary material for: Inhibition of acyl‐CoA synthetase long‐chain isozymes decreases multiple myeloma cell proliferation and causes mitochondrial dysfunction
Source: Mol Oncol. 2025 Jan 23;19(6):1687–706. doi: 10.1002/1878-0261.13794 (PMC12161464; doi:10.1002/1878-0261.13794)
Supplement: Supplementary file 1 — Fig. S1. MMRF bulk RNA‐sequencing data of CD138+ myeloma cells sorted from patient BM samples at baseline from the CoMMpass trial. Fig. S2. High ACSL1 expression in tumor cells correlates with worse overall survival for MM patients. Fig. S3. Cox regression analysis of bulk RNA‐seq data of ACSL4 (ENSG00000068366) expression in CD138‐positive cells from CoMMpass dataset. Fig. S4. Cox regression analysis of scRNA‐seq data of all ACSL family members, from CD138‐negative cells in MM patient BM from CoMMpass dataset. Fig. S5. Further Characterization of ACSLs in MM and Effects of TriC. Fig. S6. Ki‐67 and Cell Cycle Example Analyses. Fig. S7. Apoptosis and BAX Expression Example Analyses. Fig. S8. Effects of TriC on human PBMCs. Fig. S9. Supportive Data on RNA‐sequencing of MM.1S Cells Treated with TriC or Vehicle for 24 h. Fig. S10. Triacsin C induces lipid peroxidation in myeloma cells while simultaneously decreased cell viability. Table S1. Average Chronos Scores of Modified Hallmark Fatty Acid Metabolism Genes in 21 Human Myeloma Cell Lines from the Cancer Dependency Map version 22Q2. Table S2. qRT‐PCR Forward Primers. Table S3. qRT‐PCR Reverse Primers. Table S4. Top 10 Significantly Upregulated Reactome Pathways in MM.1S Cells Treated with TriC for 24 h based on RNA‐sequencing data. Table S5. Top 10 Significantly Upregulated KEGG Pathways in MM.1S Cells Treated with TriC for 24 h based on RNA‐sequencing data. Table S6. Top 10 Significantly Downregulated Reactome Pathways in MM.1S Cells Treated with TriC for 24 h. Table S7. All Significantly Downregulated KEGG Pathways in MM.1S Cells Treated with TriC for 24 h. Table S8. Significantly Changed Proteins Shared Among Overrepresented Pathways between MM.1S cells Treated with 1 or 2 μm TriC for 48 h. [file MOL2-19-1687-s001.zip › Supplementary Tables Figure legends.docx]

# SUPPLEMENTAL FIGURE LEGENDS

**Supplementary Figure 1: MMRF bulk RNAsequencing data of CD138+ myeloma cells sorted from patient BM samples at baseline from the CoMMpass trial**. Baseline (n=754 patients) mRNA gene expression is shown here for the following genes (Ensembl label): *ACSL1* (ENSG00000151726), *ACSL3* (ENSG00000123983), *ASCL4* (ENSG00000068366), *ASCL5* (ENSG00000197142), *ASCL6* (ENSG00000164398) as log_2_TPM (Transcripts Per Million) values.

**Supplementary Figure 2: High *ACSL1* expression in tumor cells correlates with worse overall survival for MM patients.** Hazard ratios for overall survival of patients from CoMMPass trial with high or low *ACSL1* expression (relative to median, ENSG00000151726) for all patients (n=754). Multivariate Cox proportional-hazards model of data from Fig 1A demonstrating that accounting for patient age, BMI (over 30), International Staging System (ISS) value, and gender does not explain *ACSL1’s* prognostic effects.

**Supplementary Figure 3: Cox-regression analysis of bulk RNAseq data of *ACSL4* (ENSG00000068366) expression in CD138-positive cells from CoMMpass dataset. A)** High *ACSL4* expression in tumor cells correlates with worse overall survival for all MM patients (n=754). **B)** High *ACSL4* expression in tumor cells correlates with worse Time to Second line treatment for all MM patients (n=754). **C)** High *ACSL4* expression in tumor cells correlates with worse Time to Second line treatment for MM patients for subgroup of patients whose first-line therapy was combined bortezomib/IMIDs-based (n=373). For A-C, P-values are calculated from log-rank Kaplan-Meier test.  **D)** Hazard Ratios from multivariate analysis of *ACSL4 effects on Overall Survival* for all patients*,* using cox regression (OS) adjusted for age, BMI, ISS, and gender (1=male, 2=female).

**Supplementary Figure 4: Cox-regression analysis of scRNAseq data of all ACSL family members, from CD138-negative cells in MM patient BM from CoMMpass dataset.** **A)** Table of hazard ratios for each significant result of a comparison of high vs low ACSL family members, along with the p value of the log-rank test of equal hazards. **B)** Kaplan-Meier plot of data from A: Patients with low (blue) *ACSL3* expression in their Naïve B cells have worse survival than patients whose Naïve B cells have high (orange) *ACSL3* expression **C)** Kaplan-Meier plot of data from A: Patients with low (blue) *ACSL6* expression in their Naïve CD8 T-cells have worse survival than patients whose Naïve CD8 T cells have high (orange) *ACSL6* expression. For B-C, p-values are calculated from log-rank Kaplan-Meier test.

**Supplementary Figure 5: Further Characterization of ACSLs in MM and Effects of TriC. A)** Relative protein expression of the ACSL family and the house keeping proteins Tata-box binding protein (TBP) and glyceraldehyde-3-phosphate dehydrogenase (GAPDH) of 6 human myeloma cell lines from the Cancer Dependency Map/Cancer Cell Line Encyclopedia. **B)** ACSL family member gene expression (transcripts per million+1 (TPM+1)) in 30 human myeloma cell lines from the Cancer Dependency Map/Cancer Cell Line Encyclopedia. Cell lines used in this study are highlighted. **C)** MM.1S cells were incubated with various doses of TriC for 24, 48 and 72 hours Trypan Blue. All live (trypan negative cells) were counted on a hemacytometer. **D)** Expression of the ACSL family members in MM.1S cells treated with vehicle, 1, or 2 μM triacin C for 48 hours; assessed by qRT-PCR; n=3. **E)** Cell cycle distribution of OPM-2 cells treated with various doses of TriC for 48 hrs and stained with DAPI; n=3. **F)** Cell cycle distribution of RPMI-8226 cells treated with various doses of TriC for 48 hrs and stained with DAPI; n=3. **G, H)** Apoptosis (Annexin V-APC/DAPI) data for MM.1S and OPM2 treated with TriC for 48 hrs; n=3. **Statistics:** (C-H) Two-way ANOVA with Tukey’s multiple comparisons test. Data are mean ± StDev, *p<0.05, **p<0.01, ***p<0.001 ****p<0.0001.

**Supplementary Figure 6: Ki-67 and Cell Cycle Example Analyses.** Representative Ki67 and cell cycle distribution flow cytometry plots depicting the gating strategies in MM.1S cells treated with various concentrations of TriC for 48 hours. An initial gate was made in the FSC-A vs. SSC-A and doublets were excluded comparing the FSC-A vs. FSC-H. In the same sample, both DAPI and Ki67-AF647 was analyzed, positive populations were identified by comparing stained and unstained samples. Representative histograms of Ki67 (top) and DAPI staining (bottom) depicting fluorescent intensities vs. normalized counts (to the mode) for various concentrations of TriC. A minimum of 10,000 events were collected.

**Supplementary Figure 7: Apoptosis and BAX Expression Example Analyses. A)** Representative apoptosis assay (Annexin V/DAPI) flow cytometry plots depicting the gating strategies in MM.1S cells treated with various concentrations of TriC for 48 hours. An initial gate was made in the FSC-A vs. SSC-A and doublets were excluded comparing the FSC-A vs. FSC-H. Within the single cell gate, positive populations were identified by comparing stained and unstained samples. Representative flow plots depicting fluorescent intensities of Annexin V vs. DAPI for various concentrations of TriC. A minimum of 10,000 events were collected. **B)** Representative BAX protein flow cytometry plots gated with a similar strategy as above, however positive signal was identified by comparing AF488 anti-BAX-stained samples to AF488 Isotype control-stained samples. A minimum of 10,000 events were collected.

**Supplementary Figure 8: Effects of TriC on human PBMCs. A)** Viability (% propidium iodide negative cells, relative to vehicle-treated cells) of human PBMCs treated for 48 hours. Dotted line indicates ED50 value, which is between 3 and 6 µM. **B-D)** Redox potential as measured by the relative luminescent signal (relative to vehicle-treated cells) from RealTime-Glo^TM^ MT Viability in human PBMCs, ATCC MM.1S and OPM-2 cells were treated with various doses of TriC or vehicle (DMSO) for 24 (B), 48 (C) and 72 hours (D), respectively. n=3. **Statistics:** (A) One-way ANOVA with Dunnett’s multiple comparisons test. (B-D) Two-way ANOVA with Tukey’s multiple comparison test. Data are the mean ± StDev, *p<0.05, **p<0.01, ***p<0.001 ****p<0.0001.

**Supplementary Figure 9: Supportive Data on RNAsequencing of MM.1S Cells Treated with TriC or Vehicle for 24 hours. A)** The mean sequence quality (Phred Score) for each sample and their associated read counts are displayed for MM.1S cells. **B)** Number of reads mapped for MM.1S cells treated either with vehicle or 1 μM TriC. **C.** RNA-Seq heatmap of sample-to-sample similarity as displayed via Euclidean distances of the transcriptional profiles of MM.1S cells treated either with vehicle (MV) or 1 μM TriC (MT) were calculated with DESeq2. Numbers associated with conditions designate different replicates. **D-E)** Reactome and KEGG Pathways associated with the significantly downregulated transcripts in TriC-treated MM.1S cells as assessed with Enrichr. **F-H)** qRT-PCR confirmation of downregulation of genes from RNA-seq related to **(F)** metastasis/oncogenesis, **(G)** *TP53* signaling, and **(H)** ferroptosis in MM.1S cells treated with vehicle or 1 μM TriC for 24hrs; n=3. **Statistics:** Unpaired Student’s t-test or Welch’s t-test. All data are mean ± StDev, *p<0.05, **p<0.01.

**Supplementary Figure 10: Triacsin C induces lipid peroxidation in myeloma cells while simultaneously decreased cell viability:** **A, B)** RealTime-Glo MT. **C, D)** Flow Cytometry MFI for BODIPY 581/591 stained MM.1S cells and OPM2 cells**. Statistics:** Two-way ANOVA with Dunnett’s multiple comparisons test. All data are the mean ± StDev, *p<0.05, **p<0.01, ***p<0.001 ****p<0.0001

# SUPPLEMENTAL TABLES

**Supplementary Table 1- Average Chronos Scores of Modified Hallmark Fatty Acid Metabolism Genes in 21 Human Myeloma Cell Lines from the Cancer Dependency Map version 22Q2.**

| Ensembl Gene ID | Gene Symbol | Gene Name | Avg Chronos Score | Std Dev |
| --- | --- | --- | --- | --- |
| ENSG00000204370 | SDHD | succinate dehydrogenase complex subunit D | -1.435 | 0.244 |
| ENSG00000143252 | SDHC | succinate dehydrogenase complex subunit C | -1.420 | 0.266 |
| ENSG00000112972 | HMGCS1 | 3-hydroxy-3-methylglutaryl-CoA synthase 1 | -1.230 | 0.379 |
| ENSG00000164032 | H2AZ1 | H2A.Z variant histone 1 | -1.201 | 0.334 |
| ENSG00000164687 | FABP5 | fatty acid binding protein 5 | -1.118 | 0.308 |
| ENSG00000198856 | OSTC | oligosaccharyltransferase complex non-catalytic subunit | -1.034 | 0.239 |
| ENSG00000126088 | UROD | uroporphyrinogen decarboxylase | -0.928 | 0.318 |
| ENSG00000073578 | SDHA | succinate dehydrogenase complex flavoprotein subunit A | -0.896 | 0.280 |
| ENSG00000100412 | ACO2 | aconitase 2 | -0.854 | 0.338 |
| ENSG00000092010 | PSME1 | proteasome activator subunit 1 | -0.852 | 0.283 |
| ENSG00000119689 | DLST | dihydrolipoamide S-succinyltransferase | -0.799 | 0.172 |
| ENSG00000080819 | CPOX | coproporphyrinogen oxidase | -0.704 | 0.250 |
| ENSG00000025770 | NCAPH2 | non-SMC condensin II complex subunit H2 | -0.614 | 0.368 |
| ENSG00000091140 | DLD | dihydrolipoamide dehydrogenase | -0.564 | 0.250 |
| ENSG00000183955 | KMT5A | lysine methyltransferase 5A | -0.530 | 0.226 |
| ENSG00000072506 | HSD17B10 | hydroxysteroid 17-beta dehydrogenase 10 | -0.521 | 0.414 |
| ENSG00000099194 | SCD | stearoyl-CoA desaturase | -0.486 | 0.297 |
| ENSG00000188690 | UROS | uroporphyrinogen III synthase | -0.365 | 0.201 |
| ENSG00000158473 | CD1D | CD1d molecule | -0.337 | 0.196 |
| ENSG00000160124 | MIX23 | mitochondrial matrix import factor 23 | -0.328 | 0.170 |
| ENSG00000068366 | ACSL4 | acyl-CoA synthetase long chain family member 4 | -0.314 | 0.361 |
| ENSG00000091483 | FH | fumarate hydratase | -0.298 | 0.144 |
| ENSG00000278540 | ACACA | acetyl-CoA carboxylase alpha | -0.295 | 0.234 |
| ENSG00000146701 | MDH2 | malate dehydrogenase 2 | -0.269 | 0.231 |
| ENSG00000117592 | PRDX6 | peroxiredoxin 6 | -0.264 | 0.174 |
| ENSG00000102172 | SMS | spermine synthase | -0.251 | 0.212 |
| ENSG00000123983 | ACSL3 | acyl-CoA synthetase long chain family member 3 | -0.229 | 0.274 |
| ENSG00000124370 | MCEE | methylmalonyl-CoA epimerase | -0.218 | 0.115 |
| ENSG00000164024 | METAP1 | methionyl aminopeptidase 1 | -0.198 | 0.212 |
| ENSG00000110090 | CPT1A | carnitine palmitoyltransferase 1A | -0.188 | 0.106 |
| ENSG00000128245 | YWHAH | tyrosine 3-monooxygenase/tryptophan 5-monooxygenase activation protein eta | -0.181 | 0.107 |
| ENSG00000122971 | ACADS | acyl-CoA dehydrogenase short chain | -0.169 | 0.188 |
| ENSG00000115255 | REEP6 | receptor accessory protein 6 | -0.160 | 0.130 |
| ENSG00000157184 | CPT2 | carnitine palmitoyltransferase 2 | -0.160 | 0.121 |
| ENSG00000205560 | CPT1B | carnitine palmitoyltransferase 1B | -0.160 | 0.111 |
| ENSG00000116882 | HAO2 | hydroxyacid oxidase 2 | -0.148 | 0.152 |
| ENSG00000131686 | CA6 | carbonic anhydrase 6 | -0.141 | 0.113 |
| ENSG00000163541 | SUCLG1 | succinate-CoA ligase GDP/ADP-forming subunit alpha | -0.136 | 0.168 |
| ENSG00000133835 | HSD17B4 | hydroxysteroid 17-beta dehydrogenase 4 | -0.130 | 0.152 |
| ENSG00000151726 | ACSL1 | acyl-CoA synthetase long chain family member 1 | -0.127 | 0.137 |
| ENSG00000083123 | BCKDHB | branched chain keto acid dehydrogenase E1 subunit beta | -0.122 | 0.138 |
| ENSG00000167315 | ACAA2 | acetyl-CoA acyltransferase 2 | -0.119 | 0.112 |
| ENSG00000198189 | HSD17B11 | hydroxysteroid 17-beta dehydrogenase 11 | -0.118 | 0.136 |
| ENSG00000169710 | FASN | fatty acid synthase | -0.118 | 0.199 |
| ENSG00000137106 | GRHPR | glyoxylate and hydroxypyruvate reductase | -0.117 | 0.099 |
| ENSG00000065833 | ME1 | malic enzyme 1 | -0.107 | 0.084 |
| ENSG00000161533 | ACOX1 | acyl-CoA oxidase 1 | -0.103 | 0.121 |
| ENSG00000109814 | UGDH | UDP-glucose 6-dehydrogenase | -0.103 | 0.185 |
| ENSG00000117305 | HMGCL | 3-hydroxy-3-methylglutaryl-CoA lyase | -0.103 | 0.100 |
| ENSG00000136143 | SUCLA2 | succinate-CoA ligase ADP-forming subunit beta | -0.101 | 0.134 |
| ENSG00000197375 | SLC22A5 | solute carrier family 22 member 5 | -0.100 | 0.087 |
| ENSG00000004961 | HCCS | holocytochrome c synthase | -0.100 | 0.123 |
| ENSG00000112033 | PPARD | peroxisome proliferator activated receptor delta | -0.099 | 0.134 |
| ENSG00000010932 | FMO1 | flavin containing dimethylaniline monoxygenase 1 | -0.095 | 0.114 |
| ENSG00000168291 | PDHB | pyruvate dehydrogenase E1 subunit beta | -0.083 | 0.205 |
| ENSG00000108515 | ENO3 | enolase 3 | -0.082 | 0.159 |
| ENSG00000095321 | CRAT | carnitine O-acetyltransferase | -0.082 | 0.102 |
| ENSG00000138413 | IDH1 | isocitrate dehydrogenase (NADP(+)) 1 | -0.081 | 0.169 |
| ENSG00000180902 | D2HGDH | D-2-hydroxyglutarate dehydrogenase | -0.080 | 0.129 |
| ENSG00000119471 | HSDL2 | hydroxysteroid dehydrogenase like 2 | -0.078 | 0.104 |
| ENSG00000138796 | HADH | hydroxyacyl-CoA dehydrogenase | -0.077 | 0.062 |
| ENSG00000104823 | ECH1 | enoyl-CoA hydratase 1 | -0.077 | 0.107 |
| ENSG00000080824 | HSP90AA1 | heat shock protein 90 alpha family class A member 1 | -0.071 | 0.113 |
| ENSG00000148090 | AUH | AU RNA binding methylglutaconyl-CoA hydratase | -0.071 | 0.110 |
| ENSG00000240972 | MIF | macrophage migration inhibitory factor | -0.070 | 0.133 |
| ENSG00000106605 | BLVRA | biliverdin reductase A | -0.068 | 0.110 |
| ENSG00000104267 | CA2 | carbonic anhydrase 2 | -0.066 | 0.154 |
| ENSG00000101473 | ACOT8 | acyl-CoA thioesterase 8 | -0.066 | 0.113 |
| ENSG00000060971 | ACAA1 | acetyl-CoA acyltransferase 1 | -0.064 | 0.094 |
| ENSG00000111674 | ENO2 | enolase 2 | -0.060 | 0.131 |
| ENSG00000116791 | CRYZ | crystallin zeta | -0.056 | 0.090 |
| ENSG00000198130 | HIBCH | 3-hydroxyisobutyryl-CoA hydrolase | -0.053 | 0.088 |
| ENSG00000213316 | LTC4S | leukotriene C4 synthase | -0.051 | 0.202 |
| ENSG00000140465 | CYP1A1 | cytochrome P450 family 1 subfamily A member 1 | -0.051 | 0.068 |
| ENSG00000143819 | EPHX1 | epoxide hydrolase 1 | -0.050 | 0.105 |
| ENSG00000144724 | PTPRG | protein tyrosine phosphatase receptor type G | -0.049 | 0.168 |
| ENSG00000005187 | ACSM3 | acyl-CoA synthetase medium chain family member 3 | -0.047 | 0.144 |
| ENSG00000115361 | ACADL | acyl-CoA dehydrogenase long chain | -0.046 | 0.090 |
| ENSG00000139112 | GABARAPL1 | GABA type A receptor associated protein like 1 | -0.042 | 0.113 |
| ENSG00000241644 | INMT | indolethylamine N-methyltransferase | -0.040 | 0.122 |
| ENSG00000186951 | PPARA | peroxisome proliferator activated receptor alpha | -0.039 | 0.084 |
| ENSG00000104320 | NBN | nibrin | -0.036 | 0.109 |
| ENSG00000138029 | HADHB | hydroxyacyl-CoA dehydrogenase trifunctional multienzyme complex subunit beta | -0.035 | 0.103 |
| ENSG00000167434 | CA4 | carbonic anhydrase 4 | -0.033 | 0.093 |
| ENSG00000164398 | ACSL6 | acyl-CoA synthetase long chain family member 6 | -0.031 | 0.121 |
| ENSG00000172340 | SUCLG2 | succinate-CoA ligase GDP-forming subunit beta | -0.031 | 0.089 |
| ENSG00000176194 | CIDEA | cell death inducing DFFA like effector a | -0.030 | 0.140 |
| ENSG00000074416 | MGLL | monoglyceride lipase | -0.028 | 0.071 |
| ENSG00000042445 | RETSAT | retinol saturase | -0.027 | 0.080 |
| ENSG00000134333 | LDHA | lactate dehydrogenase A | -0.027 | 0.144 |
| ENSG00000167969 | ECI1 | enoyl-CoA delta isomerase 1 | -0.026 | 0.111 |
| ENSG00000198721 | ECI2 | enoyl-CoA delta isomerase 2 | -0.024 | 0.116 |
| ENSG00000170835 | CEL | carboxyl ester lipase | -0.020 | 0.099 |
| ENSG00000164434 | FABP7 | fatty acid binding protein 7 | -0.017 | 0.106 |
| ENSG00000131828 | PDHA1 | pyruvate dehydrogenase E1 subunit alpha 1 | -0.013 | 0.258 |
| ENSG00000115159 | GPD2 | glycerol-3-phosphate dehydrogenase 2 | -0.012 | 0.067 |
| ENSG00000135821 | GLUL | glutamate-ammonia ligase | -0.012 | 0.131 |
| ENSG00000170323 | FABP4 | fatty acid binding protein 4 | -0.009 | 0.120 |
| ENSG00000078804 | TP53INP2 | tumor protein p53 inducible nuclear protein 2 | -0.006 | 0.117 |
| ENSG00000105679 | GAPDHS | glyceraldehyde-3-phosphate dehydrogenase, spermatogenic | -0.005 | 0.091 |
| ENSG00000012660 | ELOVL5 | ELOVL fatty acid elongase 5 | -0.004 | 0.069 |
| ENSG00000167588 | GPD1 | glycerol-3-phosphate dehydrogenase 1 | -0.001 | 0.084 |
| ENSG00000197142 | ACSL5 | acyl-CoA synthetase long chain family member 5 | -0.000 | 0.070 |
| ENSG00000104951 | IL4I1 | interleukin 4 induced 1 | 0.001 | 0.096 |
| ENSG00000109576 | AADAT | aminoadipate aminotransferase | 0.003 | 0.062 |
| ENSG00000104325 | DECR1 | 2,4-dienoyl-CoA reductase 1 | 0.005 | 0.093 |
| ENSG00000137274 | BPHL | biphenyl hydrolase like | 0.007 | 0.107 |
| ENSG00000147383 | NSDHL | NAD(P) dependent steroid dehydrogenase-like | 0.009 | 0.126 |
| ENSG00000123689 | G0S2 | G0/G1 switch 2 | 0.009 | 0.151 |
| ENSG00000115758 | ODC1 | ornithine decarboxylase 1 | 0.009 | 0.143 |
| ENSG00000197416 | FABP12 | fatty acid binding protein 12 | 0.011 | 0.147 |
| ENSG00000101365 | IDH3B | isocitrate dehydrogenase (NAD(+)) 3 non-catalytic subunit beta | 0.012 | 0.198 |
| ENSG00000089248 | ERP29 | endoplasmic reticulum protein 29 | 0.015 | 0.112 |
| ENSG00000113790 | EHHADH | enoyl-CoA hydratase and 3-hydroxyacyl CoA dehydrogenase | 0.017 | 0.091 |
| ENSG00000065057 | NTHL1 | nth like DNA glycosylase 1 | 0.018 | 0.114 |
| ENSG00000139547 | RDH16 | retinol dehydrogenase 16 | 0.018 | 0.090 |
| ENSG00000135218 | CD36 | CD36 molecule (CD36 blood group) | 0.020 | 0.094 |
| ENSG00000156587 | UBE2L6 | ubiquitin conjugating enzyme E2 L6 | 0.020 | 0.116 |
| ENSG00000121769 | FABP3 | fatty acid binding protein 3 | 0.020 | 0.049 |
| ENSG00000112299 | VNN1 | vanin 1 | 0.020 | 0.068 |
| ENSG00000127884 | ECHS1 | enoyl-CoA hydratase, short chain 1 | 0.021 | 0.119 |
| ENSG00000132196 | HSD17B7 | hydroxysteroid 17-beta dehydrogenase 7 | 0.023 | 0.123 |
| ENSG00000072778 | ACADVL | acyl-CoA dehydrogenase very long chain | 0.024 | 0.100 |
| ENSG00000154930 | ACSS1 | acyl-CoA synthetase short chain family member 1 | 0.025 | 0.098 |
| ENSG00000163586 | FABP1 | fatty acid binding protein 1 | 0.027 | 0.096 |
| ENSG00000151790 | TDO2 | tryptophan 2,3-dioxygenase | 0.029 | 0.073 |
| ENSG00000166228 | PCBD1 | pterin-4 alpha-carbinolamine dehydratase 1 | 0.030 | 0.083 |
| ENSG00000159231 | CBR3 | carbonyl reductase 3 | 0.030 | 0.178 |
| ENSG00000138696 | BMPR1B | bone morphogenetic protein receptor type 1B | 0.036 | 0.157 |
| ENSG00000159228 | CBR1 | carbonyl reductase 1 | 0.042 | 0.113 |
| ENSG00000138698 | RAP1GDS1 | Rap1 GTPase-GDP dissociation stimulator 1 | 0.042 | 0.068 |
| ENSG00000170231 | FABP6 | fatty acid binding protein 6 | 0.043 | 0.065 |
| ENSG00000119673 | ACOT2 | acyl-CoA thioesterase 2 | 0.044 | 0.090 |
| ENSG00000169169 | CPT1C | carnitine palmitoyltransferase 1C | 0.046 | 0.109 |
| ENSG00000111897 | SERINC1 | serine incorporator 1 | 0.047 | 0.108 |
| ENSG00000150787 | PTS | 6-pyruvoyltetrahydropterin synthase | 0.053 | 0.091 |
| ENSG00000196344 | ADH7 | alcohol dehydrogenase 7 (class IV), mu or sigma polypeptide | 0.055 | 0.071 |
| ENSG00000100097 | LGALS1 | galectin 1 | 0.057 | 0.087 |
| ENSG00000189221 | MAOA | monoamine oxidase A | 0.060 | 0.084 |
| ENSG00000120694 | HSPH1 | heat shock protein family H (Hsp110) member 1 | 0.060 | 0.158 |
| ENSG00000132170 | PPARG | peroxisome proliferator activated receptor gamma | 0.070 | 0.104 |
| ENSG00000103150 | MLYCD | malonyl-CoA decarboxylase | 0.074 | 0.106 |
| ENSG00000100577 | GSTZ1 | glutathione S-transferase zeta 1 | 0.078 | 0.102 |
| ENSG00000197747 | S100A10 | S100 calcium binding protein A10 | 0.080 | 0.092 |
| ENSG00000067829 | IDH3G | isocitrate dehydrogenase (NAD(+)) 3 non-catalytic subunit gamma | 0.082 | 0.110 |
| ENSG00000105607 | GCDH | glutaryl-CoA dehydrogenase | 0.087 | 0.132 |
| ENSG00000145384 | FABP2 | fatty acid binding protein 2 | 0.088 | 0.071 |
| ENSG00000117054 | ACADM | acyl-CoA dehydrogenase medium chain | 0.088 | 0.111 |
| ENSG00000014641 | MDH1 | malate dehydrogenase 1 | 0.091 | 0.111 |
| ENSG00000162365 | CYP4A22 | cytochrome P450 family 4 subfamily A member 22 | 0.092 | 0.147 |
| ENSG00000067064 | IDI1 | isopentenyl-diphosphate delta isomerase 1 | 0.096 | 0.098 |
| ENSG00000120437 | ACAT2 | acetyl-CoA acetyltransferase 2 | 0.096 | 0.117 |
| ENSG00000171503 | ETFDH | electron transfer flavoprotein dehydrogenase | 0.104 | 0.118 |
| ENSG00000072042 | RDH11 | retinol dehydrogenase 11 | 0.105 | 0.112 |
| ENSG00000116133 | DHCR24 | 24-dehydrocholesterol reductase | 0.112 | 0.108 |
| ENSG00000187048 | CYP4A11 | cytochrome P450 family 4 subfamily A member 11 | 0.115 | 0.106 |
| ENSG00000164120 | HPGD | 15-hydroxyprostaglandin dehydrogenase | 0.122 | 0.124 |
| ENSG00000134240 | HMGCS2 | 3-hydroxy-3-methylglutaryl-CoA synthase 2 | 0.124 | 0.089 |
| ENSG00000136750 | GAD2 | glutamate decarboxylase 2 | 0.140 | 0.144 |

**Supplementary Table 2- qRT-PCR Forward Primers**

| **Ensembl ID** | **Target Gene Name** | **Forward Primer (5'-3')** | **Tm (C)** |
| --- | --- | --- | --- |
| ENSG00000160179 | ABCG1 | GTCTCGCTGATGAAAGGGCT | 60.11 |
| ENSG00000278540 | ACACA | ACAACGCAGGCATCAGAAGA | 57 |
| ENSG00000151726 | ACSL1 | GTGGAACTACAGGCAACCCC | 58.2 |
| ENSG00000123983 | ACSL3 | GGAACAATTTCCGAAGTGTGGG | 56.5 |
| ENSG00000068366 | ACSL4 | CCGCCCCTCCGCACAATAA | 60.6 |
| ENSG00000197142 | ACSL5 | TGCCAAAACCAAGTCAAAGCC | 56.8 |
| ENSG00000164398 | ACSL6 | AAATCGGCCAGAGTGGATCA | 56.4 |
| ENSG00000169020 | ATP5ME | GCCACGCGCTACAATTACCT | 61.09 |
| ENSG00000128965 | CHAC1 | GTGTGGAGGCCCGACTTC | 60.05 |
| ENSG00000178741 | COX5A | TTGATGCTCGCTGGGTAACA | 59.68 |
| ENSG00000126267 | COX6B1 | CGGGGTGCCTTTAGGATTCA | 59.75 |
| ENSG00000175197 | DDIT3 | GAGCTGGAAGCCTGGTATGA | 59.17 |
| ENSG00000168209 | DDIT4 | GGTTTGACCGCTCCACGAG | 61.03 |
| ENSG00000086232 | EIF2AK1 | CAACTCCGGGGTCCGCAA | 62.32 |
| ENSG00000172071 | EIF2AK3 | GCCAATTCAATGCCTGGGAC | 59.82 |
| ENSG00000128829 | EIF2AK4 | ATAACAAGCCCCCTCCCAAG | 59.37 |
| ENSG00000178607 | ERN1 | ACCCAGAGAAGCACGAAGAC | 59.68 |
| ENSG00000167468 | GPX4 | CAGTGAGGCAAGACCGAAGT | 59.97 |
| ENSG00000178127 | NDUFV2 | CCCGCCATGTTCTTCTCCG | 60.82 |
| ENSG00000188747 | NOXA1 | TGTGGATCGTGGGGACTGG | 61.29 |
| ENSG00000137843 | PAK6 | TCCAGCCCATGAAGACAGTG | 59.67 |
| ENSG00000158828 | PINK1 | CCTCCAGACGTGAGACAGTT | 59.04 |
| ENSG00000109819 | PPARGC1A | CCAAAGGATGCGCTCTCGTTCA | 63.42 |
| ENSG00000155846 | PPARGC1B | GGCGCTTTGAAGTGTTTGGT | 59.9 |
| ENSG00000087074 | PPP1R15A | CCCTAAAGGCCAGAAAGGTGC | 61.23 |
| ENSG00000170667 | RASA4B | ATCGTGGAGGGGAAGAACCT | 60.25 |
| ENSG00000099194 | SCD | GCTGTCAAAGAGAAGGGGAGT | 59.65 |
| ENSG00000168003 | SLC3A2 | GATGGGTTCCAGGTTCGGG | 60.08 |
| ENSG00000151012 | SLC7A11 | TGTGCTGACAAATGTGGCCT | 60.47 |
| ENSG00000112592 | TBP | GTGGGGAGCTGTGATGTGAA | 59.6 |
| ENSG00000108064 | TFAM | GCTCAGAACCCAGATGCAAAA | 59.11 |
| ENSG00000141510 | TP53 | TCAGATAGCGATGGTCTGGC | 59.32 |
| ENSG00000101255 | TRIB3 | TTCGCTGACCGTGAGAGGAAG | 62.08 |

**Supplementary Table 3- qRT-PCR Reverse Primers**

| **Ensembl ID** | **Target Gene Name** | **Reverse Primer (5'-3')** | **Tm (C)** | **Product Length (bp)** |
| --- | --- | --- | --- | --- |
| ENSG00000160179 | ABCG1 | TGACTCAGGACGTAAAGCTGG | 59.73 | 110 |
| ENSG00000278540 | ACACA | GTTTCACCGCACACTGTTCC | 56.9 | 92 |
| ENSG00000151726 | ACSL1 | ATCATCTGGGCAAGGATTGAC | 55 | 113 |
| ENSG00000123983 | ACSL3 | CCCTGGGGTGTGGCTTATC | 58.2 | 127 |
| ENSG00000068366 | ACSL4 | ACAAGTGGACAGGCAGCAAAA | 57.7 | 129 |
| ENSG00000197142 | ACSL5 | TGTTGGTGTCAAGAGCCCAT | 56.8 | 75 |
| ENSG00000164398 | ACSL6 | TCATAGAGCGGGACCACCA | 58 | 72 |
| ENSG00000169020 | ATP5ME | TCTCTGGCAATCCGTTTCAGT | 59.65 | 101 |
| ENSG00000128965 | CHAC1 | ACACGGCCAGGCATCTTG | 60.36 | 119 |
| ENSG00000178741 | COX5A | ACAAGTGTGTTTATCCCTTTACGC | 59.79 | 79 |
| ENSG00000126267 | COX6B1 | GGGGCGGTCTTGTAGTTCTT | 59.68 | 72 |
| ENSG00000175197 | DDIT3 | GGTGAAGATTTTTGATTCTTCCTCT | 57.54 | 111 |
| ENSG00000168209 | DDIT4 | GGTAAGCCGTGTCTTCCTCC | 60.11 | 93 |
| ENSG00000086232 | EIF2AK1 | TTCTGCTGGAACATCAGATTCGTC | 61.15 | 127 |
| ENSG00000172071 | EIF2AK3 | TCCCGAGCCAATTCCCTATTG | 59.86 | 120 |
| ENSG00000128829 | EIF2AK4 | GCAGGATTTCACGTTGCTCC | 59.83 | 120 |
| ENSG00000178607 | ERN1 | GCTCCAGAAGAACGGGTGTT | 60.25 | 116 |
| ENSG00000167468 | GPX4 | TTACTCCCTGGCTCCTGCTT | 60.55 | 124 |
| ENSG00000178127 | NDUFV2 | ATTCCTTACATGTCTTCCCCAGT | 59.15 | 78 |
| ENSG00000188747 | NOXA1 | GGCTTGGTCAAATGCCCGC | 62.65 | 142 |
| ENSG00000137843 | PAK6 | AGGGTGTTGGAGCTGATGAC | 59.67 | 109 |
| ENSG00000158828 | PINK1 | CTCGGGCAGATGGTCTCTTG | 60.18 | 70 |
| ENSG00000109819 | PPARGC1A | CGGTGTCTGTAGTGGCTTGACT | 62.23 | 147 |
| ENSG00000155846 | PPARGC1B | CCGTACTTCTCGCCTCTCCT | 60.75 | 73 |
| ENSG00000087074 | PPP1R15A | TGCGATCCCGAGCAAGC | 60.18 | 120 |
| ENSG00000170667 | RASA4B | CACTGTGGCTGTCCTGATGA | 59.68 | 105 |
| ENSG00000099194 | SCD | AGCCAGGTTTGTAGTACCTCCT | 60.49 | 91 |
| ENSG00000168003 | SLC3A2 | CGCAATCAAGAGCCTGTCTTC | 59.6 | 108 |
| ENSG00000151012 | SLC7A11 | CGCTCAGAAAAGGTCACTGC | 59.49 | 87 |
| ENSG00000112592 | TBP | TGCTCTGACTTTAGCACCTGT | 59.31 | 183 |
| ENSG00000108064 | TFAM | GCCACTCCGCCCTATAAGC | 60.3 | 115 |
| ENSG00000141510 | TP53 | CTCATAGGGCACCACCACAC | 60.39 | 117 |
| ENSG00000101255 | TRIB3 | TTGTCCCACAGGGAATCATCTG | 60.03 | 89 |

**Supplementary Table 4- Top 10 Significantly Upregulated Reactome Pathways in MM.1S Cells Treated with TriC for 24 hours based on RNA-sequencing data**

| **Reactome Term** | **p-value** | **q-value** | **Significantly Associated Genes** |
| --- | --- | --- | --- |
| Response Of EIF2AK1 (HRI) To Heme Deficiency R-HSA-9648895 | 1.16E-13 | 5.05E-11 | [PPP1R15A, CEBPB, DDIT3, ASNS, TRIB3, CHAC1, ATF3, ATF4] |
| Regulation Of Cholesterol Biosynthesis By SREBP (SREBF) R-HSA-1655829 | 1.66E-08 | 3.61E-06 | [SREBF1, HMGCS1, SCD, INSIG1, FASN, DHCR7, SEC24D, ACACA] |
| Cellular Responses To Stress  R-HSA-2262752 | 1.45E-07 | 1.96E-05 | [PPP1R15A, KDM6B, JUN, CEBPB, CDKN1A, CBX4, EIF2AK3, ASNS, WIPI1, SLC7A11, FOS, HSPA13, VEGFA, ERN1, DDIT3, RPS6KA2, SESN2, HMOX1, TRIB3, CHAC1, ATF3, ATF4] |
| Cellular Responses To Stimuli  R-HSA-8953897 | 2.02E-07 | 1.96E-05 | [PPP1R15A, KDM6B, JUN, CEBPB, CDKN1A, CBX4, EIF2AK3, ASNS, WIPI1, SLC7A11, FOS, HSPA13, VEGFA, ERN1, DDIT3, RPS6KA2, SESN2, HMOX1, TRIB3, CHAC1, ATF3, ATF4] |
| PERK Regulates Gene Expression  R-HSA-381042 | 2.26E-07 | 1.96E-05 | [CEBPB, DDIT3, EIF2AK3, ASNS, ATF3, ATF4] |
| Unfolded Protein Response (UPR)  R-HSA-381119 | 7.59E-07 | 5.49E-05 | [ERN1, CEBPB, DDIT3, EIF2AK3, ASNS, WIPI1, ATF3, ATF4] |
| Activation Of Gene Expression By SREBF (SREBP)  R-HSA-2426168 | 1.22E-06 | 7.58E-05 | [SREBF1, HMGCS1, SCD, FASN, DHCR7, ACACA] |
| NR1H2 And NR1H3-mediated Signaling  R-HSA-9024446 | 2.12E-06 | 0.0001 | [ABCA1, MYLIP, SREBF1, SCD, FASN, ABCG1] |
| ATF4 Activates Genes In Response To Endoplasmic Reticulum Stress  R-HSA-380994 | 2.58E-06 | 0.0001 | [CEBPB, DDIT3, ASNS, ATF3, ATF4] |
| Metabolism Of Lipids  R-HSA-556833 | 3.92E-05 | 0.0016 | [ABCA1, ARHGAP9, SREBF1, CHKB, HMGCS1, INSIG1, MID1IP1, ACSL3, SGPP2, ACACA, UGCG, SCD, FASN, LPCAT3, TRIB3, DHCR7, SEC24D, ABCD1] |

**Supplementary Table 5- Top 10 Significantly Upregulated KEGG Pathways in MM.1S Cells Treated with TriC for 24 hours based on RNA-sequencing data**

| **KEGG Term** | **p-value** | **q-value** | **Significantly Associated Genes** |
| --- | --- | --- | --- |
| Ferroptosis | 0.000001 | 0.000172 | [LPCAT3, HMOX1, SLC3A2, SLC7A11, ACSL3, SAT1] |
| Lipid and atherosclerosis | 0.000002 | 0.000172 | [ERN1, ABCA1, JUN, CCL3L1, DDIT3, EIF2AK3, CCL3, FOS, LDLR, ABCG1, ATF4] |
| Apoptosis | 0.000003 | 0.000178 | [ERN1, JUN, TUBA1A, DDIT3, EIF2AK3, PMAIP1, FOS, ATF4, BBC3] |
| Parathyroid hormone synthesis, secretion and action | 0.000029 | 0.001338 | [EGR1, CDKN1A, JUND, MAFB, FOS, ATF4, CREB5] |
| Fluid shear stress and atherosclerosis | 0.000163 | 0.005457 | [JUN, DUSP1, HMOX1, FOS, ACVR2A, ASS1, VEGFA] |
| MAPK signaling pathway | 0.000179 | 0.005457 | [JUN, JUND, DUSP10, DUSP1, DDIT3, RPS6KA2, FOS, DUSP16, ATF4, VEGFA] |
| Toll-like receptor signaling pathway | 0.000232 | 0.006055 | [JUN, CCL3L1, CCL4L2, CCL4, CCL3, FOS] |
| Non-alcoholic fatty liver disease | 0.000318 | 0.00727 | [ERN1, SREBF1, JUN, DDIT3, EIF2AK3, FOS, ATF4] |
| Fatty acid biosynthesis | 0.000418 | 0.008501 | [FASN, ACSL3, ACACA] |
| AMPK signaling pathway | 0.000500 | 0.008702 | [SREBF1, SCD, FASN, ACACA, PCK2, CREB5] |

**Supplementary Table 6- Top 10 Significantly Downregulated Reactome Pathways in MM.1S Cells Treated with TriC for 24 hours**

| **Reactome Term** | **p-value** | **q-value** | **Significantly Associated Genes** |
| --- | --- | --- | --- |
| Aryl Hydrocarbon Receptor Signaling  R-HSA-8937144 | 0.0142 | 0.3086 | [ARNT2] |
| POU5F1 (OCT4), SOX2, NANOG Repress Genes Related To Differentiation R-HSA-2892245 | 0.0183 | 0.3086 | [SOX2] |
| Activation Of Ca-permeable Kainate Receptor  R-HSA-451308 | 0.0183 | 0.3086 | [GRIK4] |
| Type I Hemidesmosome Assembly  R-HSA-446107 | 0.0183 | 0.3086 | [ITGB4] |
| POU5F1 (OCT4), SOX2, NANOG Activate Genes Related To Proliferation R-HSA-2892247 | 0.0243 | 0.3086 | [SOX2] |
| Activation Of Arylsulfatases  R-HSA-1663150 | 0.0263 | 0.3086 | [ARSI] |
| Clathrin-mediated Endocytosis  R-HSA-8856828 | 0.0342 | 0.3086 | [AMPH, FNBP1L] |
| Gastrulation  R-HSA-9758941 | 0.0342 | 0.3086 | [SOX2] |
| Syndecan Interactions  R-HSA-3000170 | 0.0402 | 0.3086 | [ITGB4] |
| Laminin Interactions  R-HSA-3000157 | 0.0441 | 0.3086 | [ITGB4] |

**Supplementary Table 7- All Significantly Downregulated KEGG Pathways in MM.1S Cells Treated with TriC for 24 hours**

| **KEGG Term** | **p-value** | **q-value** | **Significantly Associated Genes** |
| --- | --- | --- | --- |
| Renal cell carcinoma | 0.0088 | 0.35041 | [ARNT2, PAK6] |
| Viral protein interaction with cytokine and cytokine receptor | 0.0178 | 0.35041 | [IL22RA1, CX3CR1] |
| Mannose type O-glycan biosynthesis | 0.0461 | 0.35041 | [B3GAT2] |

**Supplementary Table 8- Significantly Changed Proteins Shared Among Overrepresented Pathways between MM.1S cells Treated with 1 or 2 μM TriC for 48 hours**

| **UniProt ID** | **Gene Symbol** | **1 μM TriC p-value** | **1 μM TriC log2(FC)** | **2 μM TriC p-value** | **2 μM TriC log2(FC)** |
| --- | --- | --- | --- | --- | --- |
| P14854 | COX6B1 | 0.00036 | -2.4301 | 0.00196 | -3.1648 |
| P00441 | SOD1 | 0.00311 | -1.7038 | 0.0058 | -2.3216 |
| P30046 | DDT | 0.0025 | -1.649 | 0.00148 | -2.0939 |
| Q13404 | UBE2V1 | 0.00441 | -1.3851 | 0.00383 | -1.8907 |
| P14174 | MIF | 0.00439 | -1.3405 | 0.0057 | -2.0357 |
| P60174 | TPI1 | 0.0077 | -1.1878 | 4.3E-05 | -1.8122 |
| P56385 | ATP5ME | 0.03366 | -1.1303 | 0.00585 | -2.2481 |
| P25774 | CTSS | 0.02113 | -1.1126 | 8.6E-05 | -1.8819 |
| Q06323 | PSME1 | 0.01176 | -1.1088 | 0.00083 | -1.5907 |
| P20674 | COX5A | 0.01349 | -0.855 | 0.00256 | -0.8827 |
| P62136 | PPP1CA | 0.00324 | -0.7742 | 0.01815 | -0.8254 |
| Q9BWD1 | ACAT2 | 0.04915 | -0.7617 | 0.00058 | -0.9762 |
| Q9Y3D6 | FIS1 | 0.00941 | -0.7583 | 0.01764 | -0.7654 |
| P19404 | NDUFV2 | 0.00352 | -0.7359 | 0.01528 | -0.9045 |
| P63279 | UBE2I | 0.02546 | -0.715 | 0.01008 | -1.0345 |
| P14406 | COX7A2 | 0.0032 | -0.7146 | 0.02682 | -0.5887 |
| P30044 | PRDX5 | 0.0071 | -0.6596 | 0.00767 | -0.577 |
| P62979 | RPS27A | 0.03995 | -0.6181 | 0.01422 | -0.8281 |
| P32119 | PRDX2 | 0.02419 | -0.5751 | 0.00307 | -0.7292 |
| Q99497 | PARK7 | 0.03034 | -0.5744 | 0.00112 | -0.8881 |
| Q06830 | PRDX1 | 0.01014 | -0.5517 | 0.00579 | -0.6419 |
| P30041 | PRDX6 | 0.02302 | -0.4917 | 0.0104 | -0.5503 |
| P40926 | MDH2 | 0.00938 | -0.4814 | 0.00099 | -0.6454 |
| Q99459 | CDC5L | 0.04299 | -0.4597 | 0.00621 | -1.0905 |
| P67775 | PPP2CA | 0.0143 | -0.4411 | 0.01451 | -0.4875 |
| Q8NCW5 | NAXE | 0.03351 | -0.4206 | 0.04732 | -0.444 |
| Q12906 | ILF3 | 0.03873 | -0.3223 | 0.005 | -0.6077 |
| P05387 | RPLP2 | 0.03781 | -0.3036 | 0.01911 | -0.5576 |
| P27797 | CALR | 0.01821 | -0.2959 | 0.01418 | -0.3125 |
| P04406 | GAPDH | 0.04388 | -0.2738 | 0.00747 | -0.377 |
| Q8WXX5 | DNAJC9 | 0.00801 | -0.2674 | 0.0031 | -0.8543 |
| P17844 | DDX5 | 0.01053 | -0.233 | 0.01265 | -0.413 |
| P00505 | GOT2 | 0.03319 | -0.2079 | 0.0133 | -0.356 |
| P68371 | TUBB4B | 0.04662 | -0.1506 | 0.02135 | -0.1749 |
| P18124 | RPL7 | 0.04337 | 0.1003 | 0.03231 | 0.12958 |
| P46459 | NSF | 0.03706 | 0.16247 | 0.00657 | 0.27477 |
| P42765 | ACAA2 | 0.02503 | 0.19968 | 0.00956 | 0.2899 |
| P49770 | EIF2B2 | 0.00981 | 0.43057 | 0.00854 | 0.57185 |
| P50416 | CPT1A | 0.02552 | 1.02346 | 0.00218 | 1.05261 |
